# Patterns in (es)citalopram prescriptions to Medicaid and Medicare patients in the United States: the potential effects of evergreening

**DOI:** 10.3389/fpsyt.2025.1450111

**Published:** 2025-03-05

**Authors:** Luke R. Cavanah, Parita K. Ray, Jessica L. Goldhirsh, Leighton Y. Huey, Brian J. Piper

**Affiliations:** ^1^ Department of Medical Education, Geisinger College of Health Sciences, Geisinger, Scranton, PA, United States; ^2^ Behavioral Health Initiative, Geisinger College of Health Sciences, Geisinger, Scranton, PA, United States; ^3^ Center for Pharmacy Innovation and Outcomes, Geisinger, Danville, PA, United States

**Keywords:** antidepressants, anxiety, cost, depression, pharmacoepidemiology, QT prolongation, SSRIs, utilization

## Abstract

**Introduction:**

Citalopram and escitalopram are among the most used medications and are key treatments for many psychiatric disorders. Previous findings suggest citalopram and escitalopram prescription rates are changing because of the patent for citalopram ending as opposed to evidence of a clear therapeutic advantage—so-called “evergreening”. This retrospective study focuses on characterizing the chronologic and geographic variation in the use of citalopram and escitalopram from 2015 to 2020 among US Medicaid and Medicare patients. We hypothesized that prescription rates of citalopram will decrease with a concurrent increase in escitalopram, consistent with “evergreening”.

**Methods:**

Citalopram and escitalopram prescription rates and costs per state were obtained from the Medicaid State Drug Utilization Database and Medicare Provider Utilization and Payment Data. States’ annual prescription rates outside a 95% confidence interval were considered significantly different from the average.

**Results:**

Overall, a decreasing trend for citalopram and an increasing trend for escitalopram prescription rates were noted in both Medicare and Medicaid patients. The differences between generic and brand were noted for both drugs, with generic forms being less expensive than the brand-name version.

**Discussion:**

Despite limited evidence suggesting that citalopram and escitalopram have any meaningful differences in therapeutic or adverse effects, there exists a noticeable decline in the use of citalopram that cooccurred with an increase in escitalopram prescribing, consistent with our hypothesis. Moreover, among these general pharmacoepidemiologic trends exists significant geographic variability. There was disproportionate spending (relative to their use) on the brand versions of these medicines relative to their generic forms.

## Introduction

Citalopram and escitalopram are two of the fifty most commonly prescribed medications in the US (1). Citalopram and escitalopram were FDA-approved for the treatment of major depressive disorder (MDD), and escitalopram is additionally FDA-approved for the treatment of generalized anxiety disorder (GAD). Both medications are used off-label for numerous other psychiatric conditions, such as obsessive-compulsive disorder, panic disorder, post-traumatic stress disorder, premenstrual dysphoric disorder, and social anxiety disorder (2). Citalopram, which is available in generic form and as Celexa ^®^, was introduced in 1998, and it is a racemic mixture of the S(+)-enantiomer (escitalopram) and the R(-)-enantiomer (R-citalopram) (2, 3). Although both citalopram and escitalopram are members of the “selective” reuptake inhibitors class, these agents showed significant, and equivalent, affinity for the sigma_1_ receptors (4). Citalopram’s affinity for the histamine (H_1_) receptor (257 ± 2.8 nm) was described as “weak” which is only slightly greater than that of escitalopram’s (1,500 ± 780 nm) (4, 5). Some studies suggest that the S-enantiomer, but not the R-enantiomer, was responsible for the therapeutic effect of citalopram (6, 7). Escitalopram and citalopram generally produced equivalent effects on six animal models of depression, anxiety, and aggression although citalopram was slightly more potent (4). Moreover, there is some evidence that perhaps the R-enantiomer results in a slightly diminished therapeutic effect of the S-enantiomer, leading to the introduction of escitalopram in 2002 (4). Escitalopram is now available in generic form and as Lexapro ^®^. The general conclusion from both basic and clinical research was that the differences between citalopram and escitalopram were of potency and not efficacy.

Part of the reason citalopram and escitalopram are so commonly used is due to their relatively high tolerability. Both patients taking citalopram and escitalopram commonly report adverse effects of dry mouth, headache, nausea and vomiting, diarrhea, insomnia, and sexual dysfunction (8–10). Rarely, these medicines may result in serious conditions, such as syndrome of inappropriate antidiuretic hormone or serotonin syndrome (8, 9). Interestingly, patients taking citalopram may have adverse effects of QTc prolongation at a rate, albeit low, that was mildly greater than patient who take escitalopram (11).

Citalopram and escitalopram are frequently used among Medicaid and Medicare patients, emphasizing the importance of investigation of these medications within these populations. Notably, psychopharmaceuticals comprised the second most prescribed medication type for outpatient Medicaid patients (12). Psychological disorders result in a huge economic burden that is estimated to continue to increase (13). Furthermore, mental disorders are one of the most commonly treated disorders within the top five percent of spenders (14). Recent research has begun to elucidate how the COVID-19 pandemic has contributed to increased frequency of these disorders (15–18), again underscoring the need to understand the pharmacoepidemiology of treatments, especially those as ubiquitous as citalopram and escitalopram. Further, recent pharmacoepidemiologic investigations have found that there have been significant state variation in prescribing patterns of multiple psychoactive medications (19–21), including other SSRIs like paroxetine (22), among US Medicare and Medicaid patients.

“Evergreening” refers to the deceptive techniques, such as incremental drug modification, that pharmaceutical companies use to continue their monopoly over a drug’s rights (23–25). There is limited evidence that supports that citalopram is a more effective or tolerable alternative to escitalopram, yet an earlier meta-analysis suggests that citalopram/escitalopram prescription rates follow the patterns that would be expected for the case of evergreening (26).

The purpose of this study was to examine patterns in citalopram and escitalopram prescription rates throughout the US among Medicaid and Medicare patients. We hypothesized that citalopram prescription rates will decrease across the time interval with a concurrent increase in escitalopram prescription rates for both Medicaid and Medicare populations. This finding would suggest that evergreening has continued to be observed with these medications. Although data comparing the efficacy and adverse effects of citalopram versus escitalopram exists, research on long-term prescribing trends and the lasting impact of evergreening beyond the patent expiration of the new drug remains limited (26). This report provides more detail on the effects of pharmaceutical development strategies like evergreening affect prescribing behaviors and the healthcare system. A secondary objective was to examine state-level disparities in the use of these antidepressants, which expands on a recent pharmacoepidemiologic report that found large state variation in the use of the SSRI paroxetine (22).

## Methods

### Procedures

Citalopram and escitalopram prescription rates and costs were obtained for Medicaid and Medicare. Medicaid prescriptions were defined as number of prescriptions dispensed as outpatient drug claims (27). Medicaid spending was defined as the total amount reimbursed to pharmacies or other providers (27) for the medication. Medicare prescriptions were defined as number of Medicare Part D claims, which include original prescriptions and refills (28). Medicare spending was defined as the total amount paid by the Part D plan, Medicare beneficiary, government subsidies, and third-party payers (28).

Medicaid and Medicare data were assessed quarterly and annually (2015-2020), respectively, due to the information publicly available. These were the most recent data at the time of data extraction. We evaluated the Medicaid State Drug Utilization database (27) and Medicare Provider Utilization and Payment Data (28) for citalopram and escitalopram prescription rates and spending per state. We evaluated the Medicaid National Drug Utilization database for citalopram and escitalopram national prescription rates per quarter. These databases have been used in numerous prior pharmacoepidemiologic reports (19–22). Prescription rates were reported per thousand enrollees to account for geographic differences in prescriptions owed to different population sizes. Procedures were defined as exempt by the Geisinger IRB.

### Data analysis

Patterns in the number of national prescriptions of generic, brand, and their sums were compared for both citalopram and escitalopram. One-sample z-tests were conducted to determine if any states’ annual prescription rates of either of these medications were significantly different from the state average for that respective year for the respective program. These procedures and data analysis techniques have been used to examine the chronological and geographic variation of prescribing patterns of other psychoactive medications, such as buprenorphine, dronabinol, cannabidiol, ketamine, esketamine, and paroxetine (19–22). The ratio of total Medicaid spending and Medicare spending for generic versus brand of these medications was also calculated. Average costs per prescription of citalopram and escitalopram were also assessed. Pearson’s correlation coefficients between population-corrected number of prescriptions of citalopram and escitalopram within the Medicaid and Medicare systems were calculated for all years examined, with p<0.05 being considered statistically significant. Cohen’s recommendations for interpretation of the effect size of Pearson’s *r* were used: 0.10, 0.30, and 0.50 represented small, medium, and large effect sizes, respectively (29). We analyzed the data using Excel and constructed figures using GraphPad Prism and Heatmapper (30).

## Results

### Medicaid

**Figure 1:**
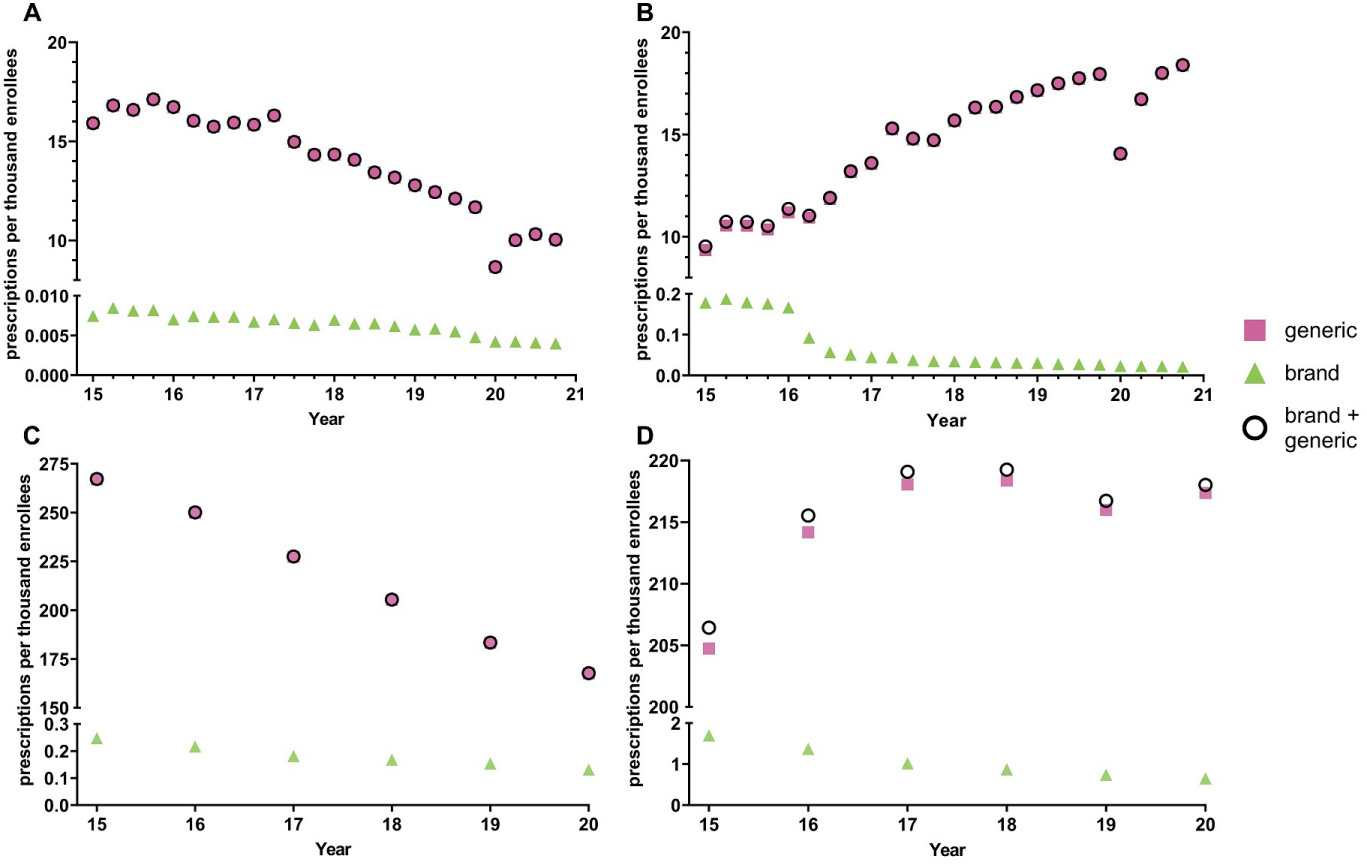
National Medicaid **(A, B)** and Medicare **(C, D)** population-corrected prescriptions for citalopram **(A, C)** and escitalopram **(B, D)** 2015-2020.

**Figure 1A** shows -26.0% decreasing prescriptions for citalopram from 2015 to 2019 and +41.2% increasing rate of prescriptions for escitalopram. The brand names for both citalopram (-42.3%) and escitalopram (-85.9%) had a decreasing rate of prescriptions from 2015 to 2020. Analysis of generic and brand name prescriptions combined revealed similar rates of prescriptions as their respective generic version amongst Medicaid patients.

Quarterly examination revealed a decrease in prescription rates in the first quarter of 2020 relative to the fourth quarter of 2019, a -25.9% reduction for citalopram and -21.7% reduction for escitalopram, followed by an increase in prescription rates for both citalopram and escitalopram during the later quarters. Prescriptions for the brand names of the respective drugs also showed a gradual decrease throughout the years, except for Lexapro, which displayed a -55.6% decrease in the first quarter of 2016 (**Figure 1B**). Overall, the prescribing rates for the brand name of the respective drugs were significantly lower compared to its generic counterpart (**Figures 1A, B**). Analysis of cost per prescription data shows an average of a 50-fold increase in the price for the brand names of citalopram and escitalopram compared to its generic counterparts.

**Figure 2:**
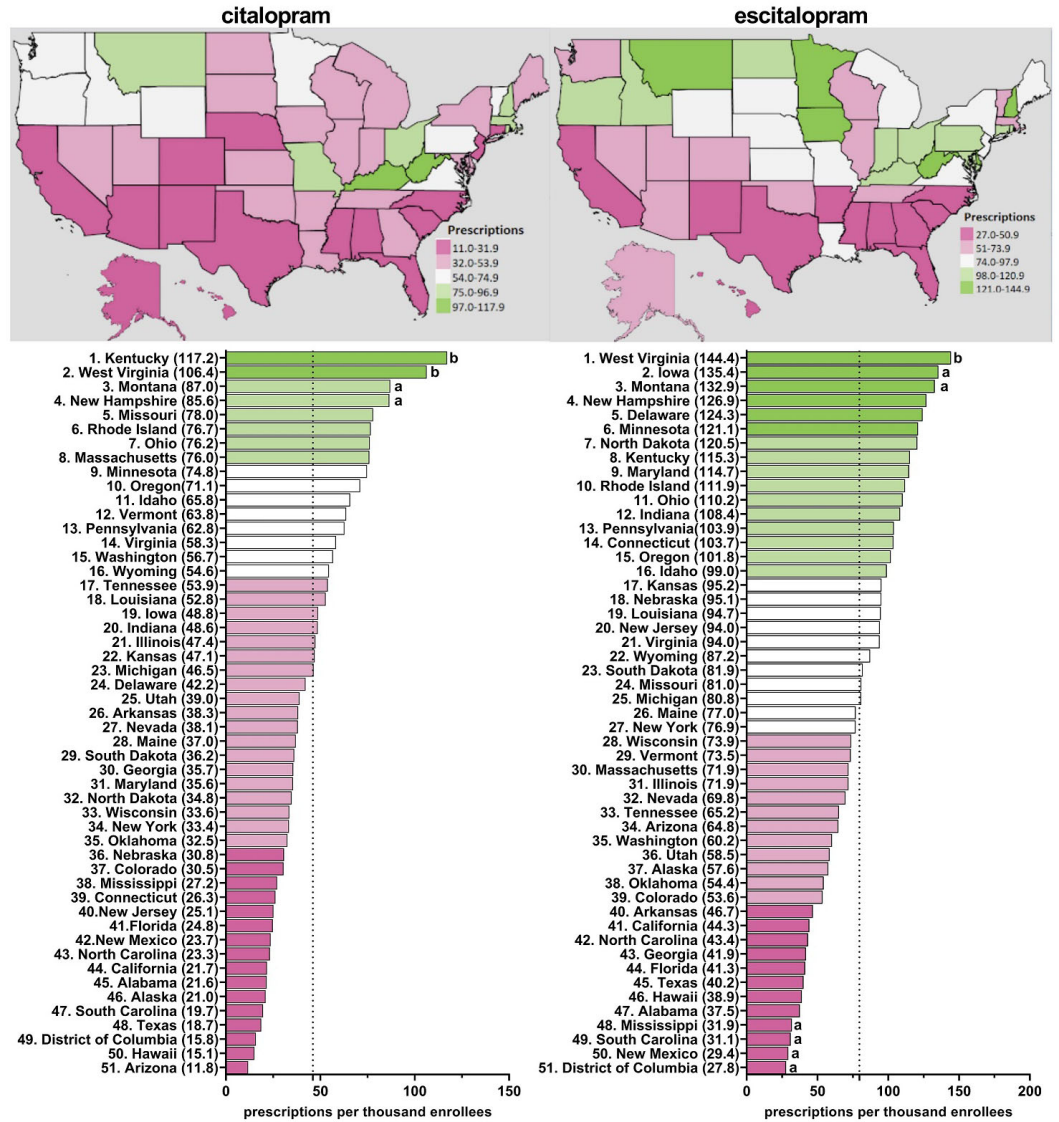
Citalopram and escitalopram prescriptions per thousand Medicaid enrollees heatmap (top) and population-corrected prescription rate per state (bottom) in 2020. ^a^indicates >1.50 SD (24.0) from the mean (46.1). ^b^indicates >1.96 SD from the mean.

**Supplementary Table 1** shows pronounced disparities by state. On average, there was a nineteen-fold difference between the highest and lowest states for escitalopram relative to an eleven-fold difference for citalopram. **Figure 2** and **Supplementary Figures 1**-**5** display state-level variations in the prescription for citalopram amongst Medicaid patients. In 2020, Kentucky (117.2), the highest prescribing state, was 9.9-fold greater than Arizona (11.8), the lowest prescribing state (**Figure 2**). Kentucky (117.2) and West Virginia (106.4) showed statistically significant (p<0.05) higher rates of prescriptions compared to the national average (**Figure 2**). Kentucky and West Virginia continued to be statistically significantly higher in the rates of prescriptions compared to the national average for 2016, 2017, and 2018 (**Supplementary Figures 2**–**4**). For 2015 and 2019, only West Virginia (155.8) was statistically significantly higher compared to the national average (**Supplementary Figure 5**).

**Figure 2** and **Supplementary Figures 6**–**10** display state-level variations in the prescription for escitalopram amongst Medicaid patients. In 2020, West Virginia (144.4), the highest prescribing state, was 5.2-fold greater compared to the lowest prescribing area, the District of Columbia (27.8) (**Figure 2**). West Virginia was consistently among the highest statistically significant prescribing states of escitalopram from 2015-2020 (**Figure 2**, **Supplementary Figures 6**–**10**). In 2018, North Dakota was also among the top prescribers of escitalopram (**Supplementary Figure 9**). Additionally, in 2015 and 2016, Connecticut and Iowa were among the highest statistically significant prescribing states compared to the national average (**Supplementary Figures 6**–**7**).

### Medicare

**Figures 1C, D** displays a –24.5% decreasing rate of prescriptions for citalopram from 2015 to 2020 and +5.61% increasing rate of prescriptions for escitalopram for Medicare patients. The brand name versions of these drugs showed a gradual decrease in prescription rates. Like the Medicaid patient prescriptions, analysis of generic and brand name prescriptions combined revealed similar rates of prescriptions as their respective generic versions in Medicare recipients.

**Figure 3:**
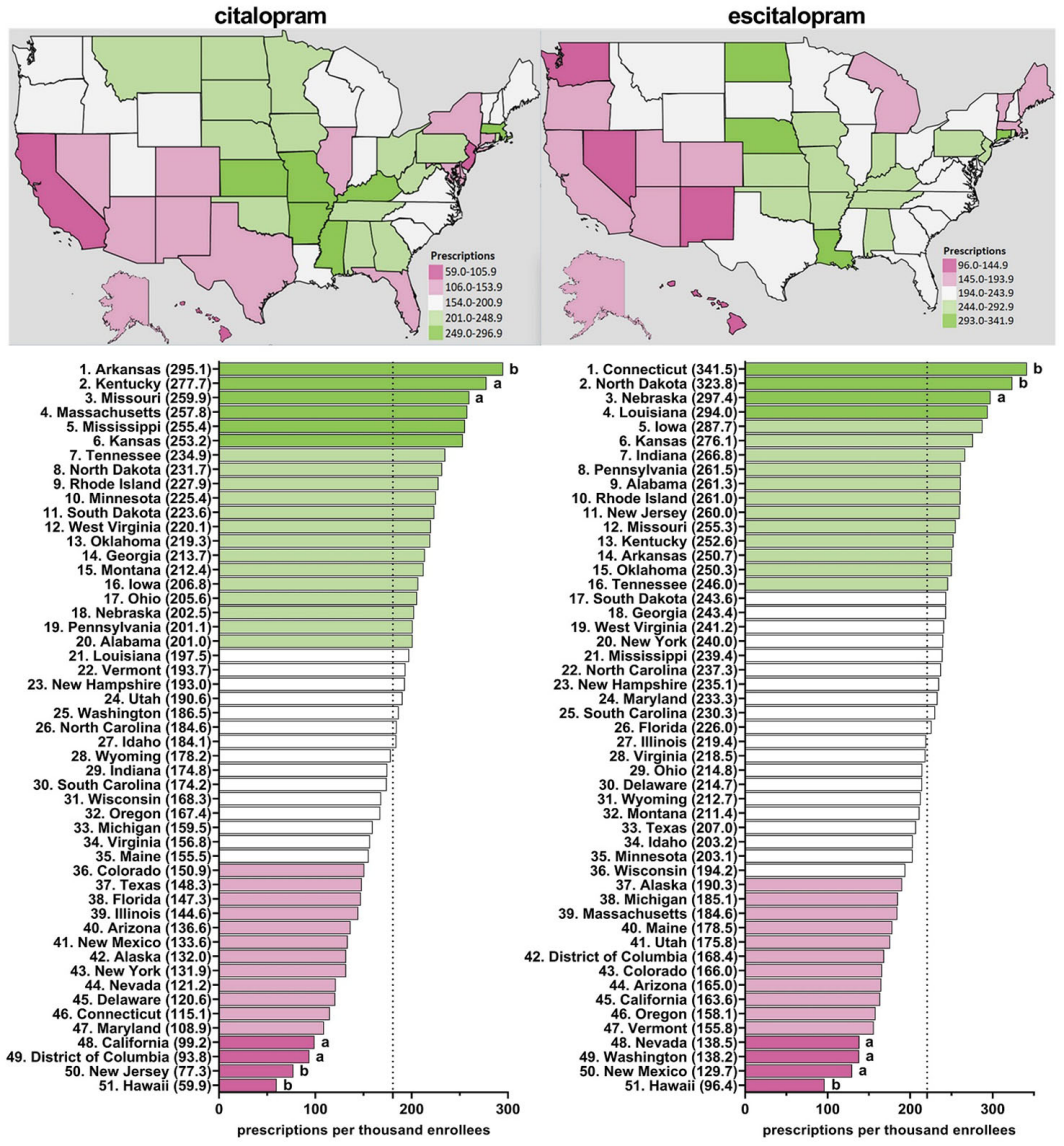
Citalopram and escitalopram prescriptions per thousand Medicare enrollees heatmap (top) and population-corrected prescription rate per state (bottom) in 2020. ^a^indicates >1.50 SD (51.8) from the mean (180.6). ^b^indicates >1.96 SD from the mean.

**Supplementary Table 1** shows appreciable (three to four-fold) state-level differences between the highest (Arkansas and Connecticut) and lowest (Hawaii) states. **Figure 3**, **Supplementary Figures 11**–**15** depict state-level variations in the prescription for citalopram amongst Medicare enrollees. From 2015-2020, Arkansas was the leading prescribing state of citalopram, with it being statistically significantly higher in the rates of prescriptions compared to the national average. Conversely, Hawaii has statistically been the lowest prescribing state compared to the national average from 2015-2020 (**Figure 3**, **Supplementary Figures 11**–**15**). In addition, New Jersey was statistically the lowest prescribing state of citalopram compared to the national average from 2015-2018 and 2020 (**Figure 3**, **Supplementary Figures 11**–**14**).

**Figure 3**, **Supplementary Figures 16**–**20** display state-level variations in the prescription for escitalopram amongst Medicare enrollees. From 2015-2020, Connecticut has consistently been the top prescriber of escitalopram and statistically higher compared to the national average (**Figure 3**, **Supplementary Figures 16**–**20**). Additionally, North Dakota was statistically higher compared to the national average in 2019 and 2020 (**Figure 3**, **Supplementary Figure 20**). Conversely, Hawaii was statistically the lowest prescriber of escitalopram compared to the national average from 2016-2020 (**Figure 3**, **Supplementary Figures 17**–**20**).

### Correlations

**Table 1** shows a moderately high correlation within Medicaid for prescribing rates of citalopram with escitalopram in 2020. The correlation between citalopram and escitalopram within Medicare was moderate and significant. For 2015-2019, **Supplementary Figures 21**–**25** displayed similar findings among the prescribing rates for both citalopram and escitalopram.

**Table 1:** Matrix of Pearson’s correlation coefficients between population-corrected number of prescriptions of citalopram and escitalopram within the Medicaid and Medicare systems for 2020 (*N*=51: 50 states and D.C.). * indicates *p ≤* 0.05, ** indicates *p ≤* 0.01, and *** *p ≤* 0.001.

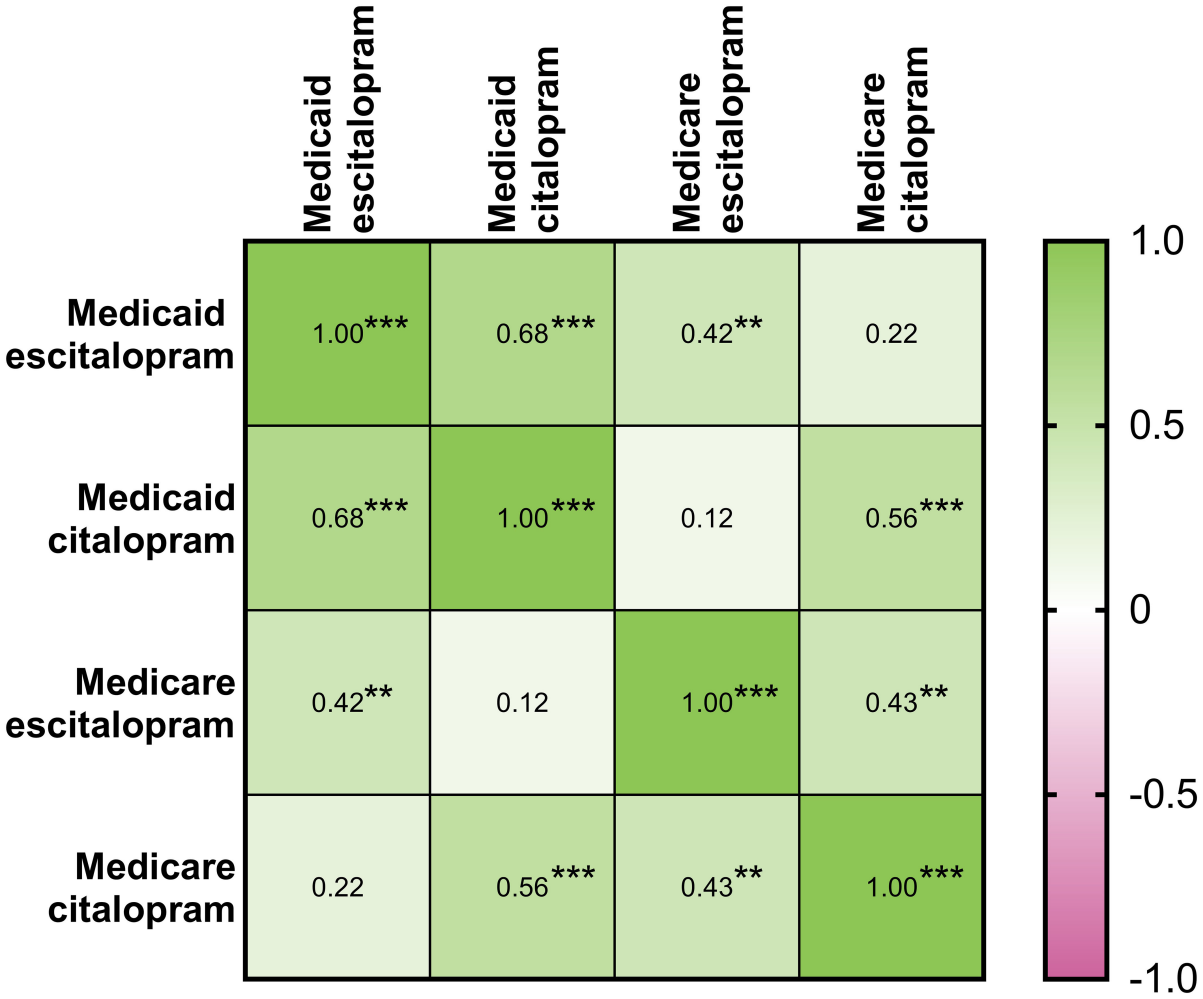

## Discussion

Between 2015-2020, the US demonstrated an increase in prescriptions of escitalopram and a corresponding decrease in prescriptions of citalopram amongst both Medicare and Medicaid patients. Despite both citalopram and escitalopram having equal efficacy (4) and similar adverse effects (8–10), we identified declining rates of prescription of citalopram. Analysis of the Medical Expenditure Panel Survey revealed 30.2 million prescriptions for citalopram and 17.0 million for escitalopram in 2013 (31). This pattern had reversed to 18.5 million citalopram prescriptions and 30.6 million escitalopram prescriptions in 2020 (1) which is congruent with the present findings. About one-fifth (19.7%) of the US gross domestic product in 2020 was spent on health care (32) and this country was an outlier relative to other developed countries (33). Examining the patterns in rates of prescription of these commonly prescribed medications provides us with an insight into evergreening and how it affects the pharmacological industry and the pharmacoepidemiology of treatments.

Additionally, we found pronounced differences in prescriptions rates of citalopram and escitalopram at the state-level. Anxiety and depressive disorders are the most common psychiatric conditions (34). An assumption of this study was that the prevalence of anxiety and depression would show relatively modest differences based on state of residence (e.g., between Connecticut and adjacent Rhode Island). However, prescriptions for escitalopram in 2015 were eighty-fold more common in Connecticut relative to Rhode Island for Medicaid. Further, Kentucky was frequently among the highest, and statistically elevated, prescriber of citalopram and escitalopram to both Medicaid and Medicare enrollees, along with West Virginia, Connecticut, and Arkansas. These findings extend upon earlier findings which found five-fold state level differences among Medicare patients receiving paroxetine (22), six-fold for prescription stimulants (35), and twenty-fold for meperidine (36).

Escitalopram was initially introduced in 2002 as the S-enantiomer, while citalopram was introduced four years earlier in 1998 (2–4). Clinical trials have determined that both escitalopram and citalopram significantly improve symptoms of depression and anxiety (37) but that escitalopram may have a quicker onset and an overall greater effect on improving symptoms of anxiety and depression compared to citalopram among patients with major depressive disorder (37). Although unlikely, this may be a possible explanation for the rise of escitalopram use over citalopram. The landmark Sequenced-Treatment-Alternatives to Relieve Depression (STAR*D) which found only a one-third remission rate with citalopram (38) may have tempered enthusiasm for the treatment of depression. Another possible explanation for the observed change in preference from citalopram to escitalopram is the evidence that citalopram has a higher likelihood of QT prolongation (11). Additionally, the FDA noted the concern about QT prolongation with citalopram use which may have influenced physicians to prescribe escitalopram over citalopram (39). While major drawbacks with SSRIs are the therapeutic lag and a black box warning for increased risk of suicidality among young-adults, further study will be needed to determine if novel agents like esketamine (19) will one day begin to supplant citalopram/escitalopram for depression.

Prescription rates of escitalopram in Medicaid enrollees almost doubled from 2015 to 2020 which may be explained by evergreening. Typically, the evergreened drug is released into the market before the patent expiration of the original drug. To compete with the original drug, one would expect the prices of the drugs to be competitive. We discovered that the cost of escitalopram per prescription was two-times higher compared to the cost of citalopram amongst Medicaid enrollees. For Medicare enrollees, the cost of escitalopram was five times higher than citalopram. The difference in costs with respect to the trends noted in the prescriptions of these medications suggests an alternate reason, which can be further assessed by further study.

It is likely that the FDA’s.warnings,along with subsequent electronic prescribing safeguards, have also played a significant role in the observed shift from citalopram to escitalopram, beyond concerns of evergreening. Additionally, the aging Medicare population presents another critical factor influencing this transition. Given the reduced maximal dose recommendations for citalopram in older adults, clinicians may have opted for escitalopram due to its perceived safety and efficacy in this demographic. Future analyses should further explore the interplay between prescribing policies, electronic health record alerts, and the aging patient population to better contextualize our findings. Expanding the discussion on these factors will provide a more comprehensive understanding of the motivations behind prescribing shifts and improve the generalizability of our conclusions.

In general, the use of SSRIs and total spending on them has increased from 1991 to 2018 (40). The increased utilization of these drugs can also be explained by the rising prevalence of depression and the number of patients who seek pharmacological treatment. Generics were first introduced in 2001, which caused a shift in Medicaid to start the utilization of generic drugs over brand names (40). This change allowed for a significant reduction in costs of these drugs and this pattern was noted in our study as well. It is also noteworthy that prescriptions of both citalopram and escitalopram to Medicaid patients showed transient, but appreciable (-21.7 – 25.9%) reductions during the initial period of the COVID-19 pandemic. Examining effects of COVID-19 pandemic on prescribing patterns of these medications and other psychotropics using other datasets is a meaningful future direction.

### Limitations

One of the primary limitations of our retrospective study design of pre-existing prescribing data includes the inability to control confounding variables in real-time and hence cannot establish causation, but only association. To mitigate the limitations of our retrospective methodology, we implemented rigorous data validation techniques, including standardizing data extraction criteria to reduce inconsistencies. Despite these efforts, we acknowledge that a prospective study would provide a more robust framework for confirming our findings and guiding future research directions.

Although Medicaid and Medicare are large programs, these findings may not generalize to patients with private insurance or those without insurance (1). Patient populations often have distinct demographic and socioeconomic characteristics, including higher rates of chronic illnesses, disabilities, and lower-income status, which may influence healthcare utilization patterns and treatment outcomes. Future investigations into those with private insurance or without insurance merits consideration. Further, the dataset analyzed does not include age, gender, socioeconomic status, or other demographic characteristics of the patients for whom these medications were prescribed. Future studies where these analyses could be performed would provide a more comprehensive understanding of the findings.

Our study’s follow-up period, while sufficient for capturing short- to mid-term prescribing trends, may not fully encompass long-term patterns, particularly in relation to medication adherence, treatment efficacy, and evolving prescribing guidelines. Given that healthcare policies, provider practices, and patient behaviors change over time, a longer follow-up period could provide deeper insights into sustained trends and potential long-term outcomes associated with prescribing patterns. Future studies could consider a more extended follow-up period to better assess the durability of these trends, evaluate long-term patient outcomes, and identify shifts in clinical practice.

Research with other data-sources will be necessary to determine if there are state-level differences in the access and utilization of evidence-based non-pharmacological treatments for anxiety (41) and depression (42). Third, the decline in prescriptions per enrollee in 2020 among Medicaid (**Figures 1A, B**), but not Medicare (**Figures 1C, D**), could largely be due to an elevation in enrollment during COVID (43).

## Conclusion

In conclusion, from 2015 to 2020, citalopram use among US Medicaid and Medicare patients has decreased while escitalopram use continues to rise. The use of these SSRIs has also greatly shifted from brand to generic, which may be due to the high cost of brand-named drugs. Profound state-level variations in the prescriptions of these medications were noted in both Medicare and Medicaid patients. Future studies can explore the rising trends of these medications and explain the reason for the substantial state level differences among prescriptions.

## Data Availability

Publicly available datasets were analyzed in this study. This data can be found here: https://data.cms.gov/provider-summary-by-type-of-service/medicare-part-d-prescribers https://www.medicaid.gov/medicaid/prescription-drugs/state-drug-utilization-data/index.html.
