# Supplementary material for: Patterns in (es)citalopram prescriptions to Medicaid and Medicare patients in the United States: the potential effects of evergreening
Source: Front Psychiatry. 2025 Mar 5;16:1450111. doi: 10.3389/fpsyt.2025.1450111 (PMC11919847; doi:10.3389/fpsyt.2025.1450111)

**Supplemental Table 1.** Pronounced state level disparities in Medicaid and Medicare prescriptions, per 1,000 enrollees, for citalopram and escitalopram. Abbreviations for the highest and lowest states are in superscript (AR: Arkansas, AZ: Arizona, CT:

Connecticut, DC: District of Columbia, KY: Kentucky, NM: New Mexico, RI: Rhode Island, SC: South Carolina, WV:

West Virginia).

__________________________________________________________________________________________________

citalopram escitalopram

Medicaid Highest Lowest Ratio Highest Lowest Ratio

2015 167.3^WV^ 7.3^RI^ 22.9 122.7^CT^ 1.5^RI^ 81.8

2016 160.6^WV^ 22.3^HI^ 7.2 128.5^WV^ 19.2^SC^ 6.7

2017 178.6^KY^ 23.6^DC^ 7.6 132.7^WV^ 24.8^DC^ 5.4

2018 178.5^KY^ 20.4^DC^ 8.8 145.4^WV^ 24.5^DC^ 5.9

2019 152.4^KY^ 18.3^SC^ 8.3 155.8^WV^ 26.7^NM^ 5.8

2020 117.2^KY^ 11.8^AZ^ 9.9 144.4^WV^ 27.8^DC^ 5.2

Mean 163.5 17.3 10.8 138.3 20.8 18.5

Medicare

2015 453.4^AR^ 100.2^HI^ 4.5 361.4^CT^ 109.0^HI^ 3.3

2016 436.2^AR^ 92.1^HI^ 4.7 367.7^CT^ 107.7^HI^ 3.4

2017 403.2^AR^ 80.2^HI^ 5.0 365.5^CT^ 103.7^HI^ 3.5

2018 369.6^AR^ 75.0^HI^ 4.9 361.6^CT^ 99.8^HI^ 3.6

2019 328.4^AR^ 64.1^HI^ 5.1 351.4^CT^ 95.2^HI^ 3.7

2020 295.1^AR^ 59.9^HI^ 4.9 341.5^CT^ 96.4^HI^ 3.5

Mean 381.0 78.6 4.9 358.2 102.0 3.5

___________________________________________________________________________________________

**Supplemental Figure 1.** Citalopram prescriptions per thousand Medicaid enrollees heatmap (left) and population-corrected prescription rate per state (right) in 2015. ^a^ indicates >1.50 SD (32.7) from the mean (71.2). ^b^ indicates >1.96 SD from the mean.

**
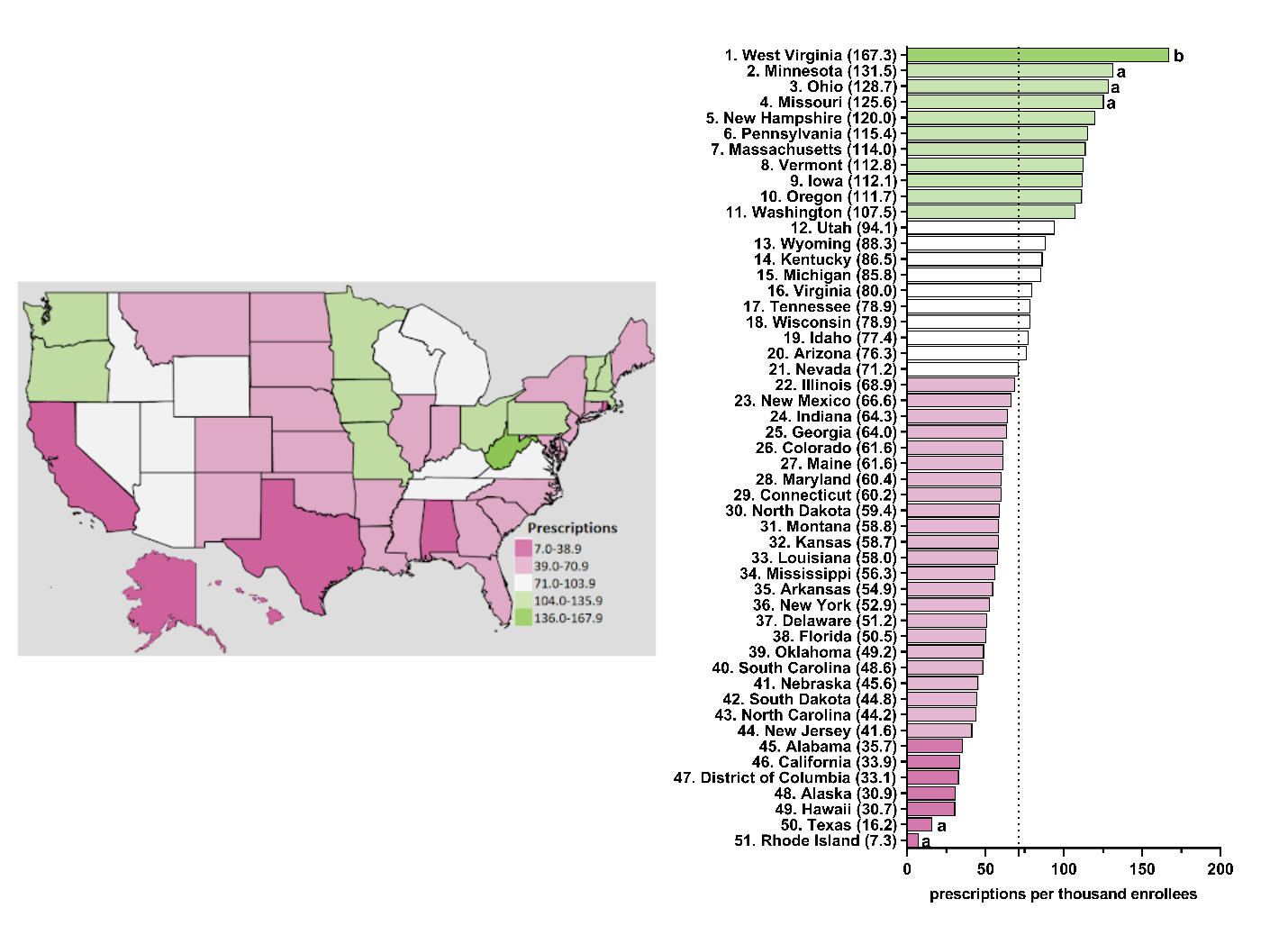
**

**Supplemental Figure 2.** Citalopram prescriptions per thousand Medicaid enrollees heatmap (left) and population-corrected prescription rate per state (right) in 2016. ^a^ indicates >1.50 SD (33.3) from the mean (71.6). ^b^ indicates >1.96 SD from the mean.


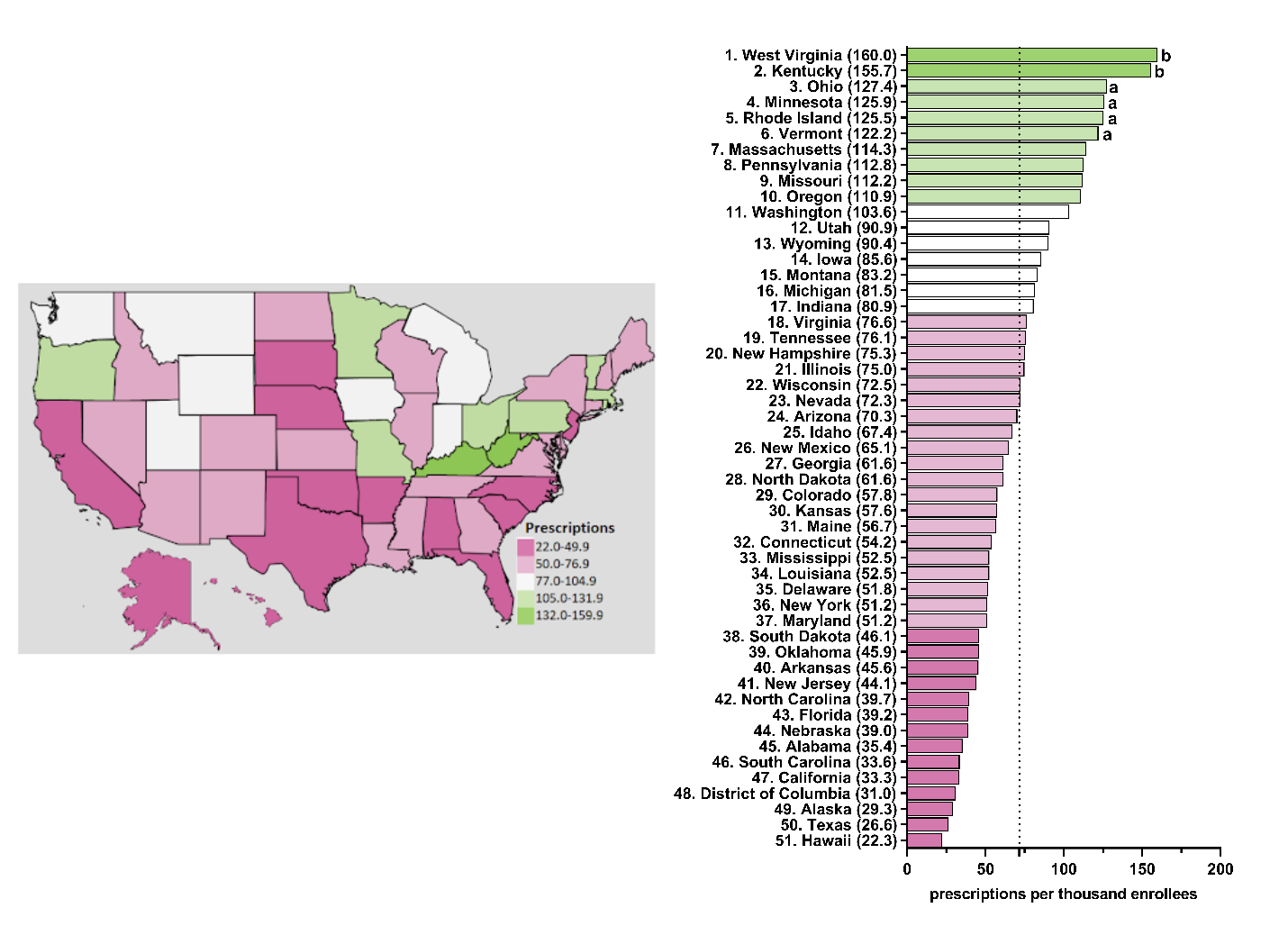


**Supplemental Figure 3.** Citalopram prescriptions per thousand Medicaid enrollees heatmap (left) and population-corrected prescription rate per state (right) in 2017. ^a^ indicates >1.50 SD (33.4) from the mean (67.4). ^b^ indicates >1.96 SD from the mean.


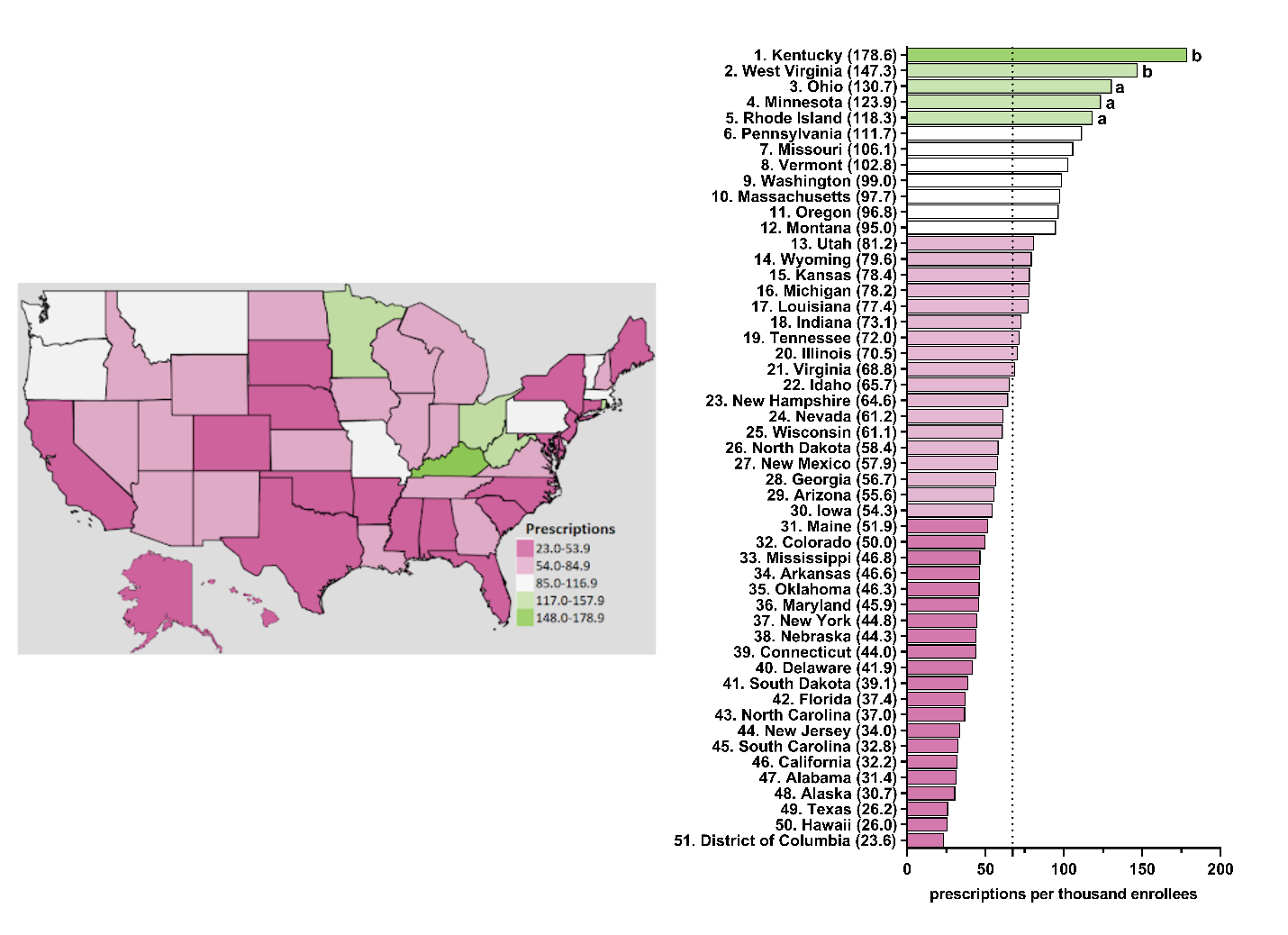


**Supplemental Figure 4.** Citalopram prescriptions per thousand Medicaid enrollees heatmap (left) and population-corrected prescription rate per state (right) in 2018. ^a^ indicates >1.50 SD (32.1) from the mean (61.0). ^b^ indicates >1.96 SD from the mean.


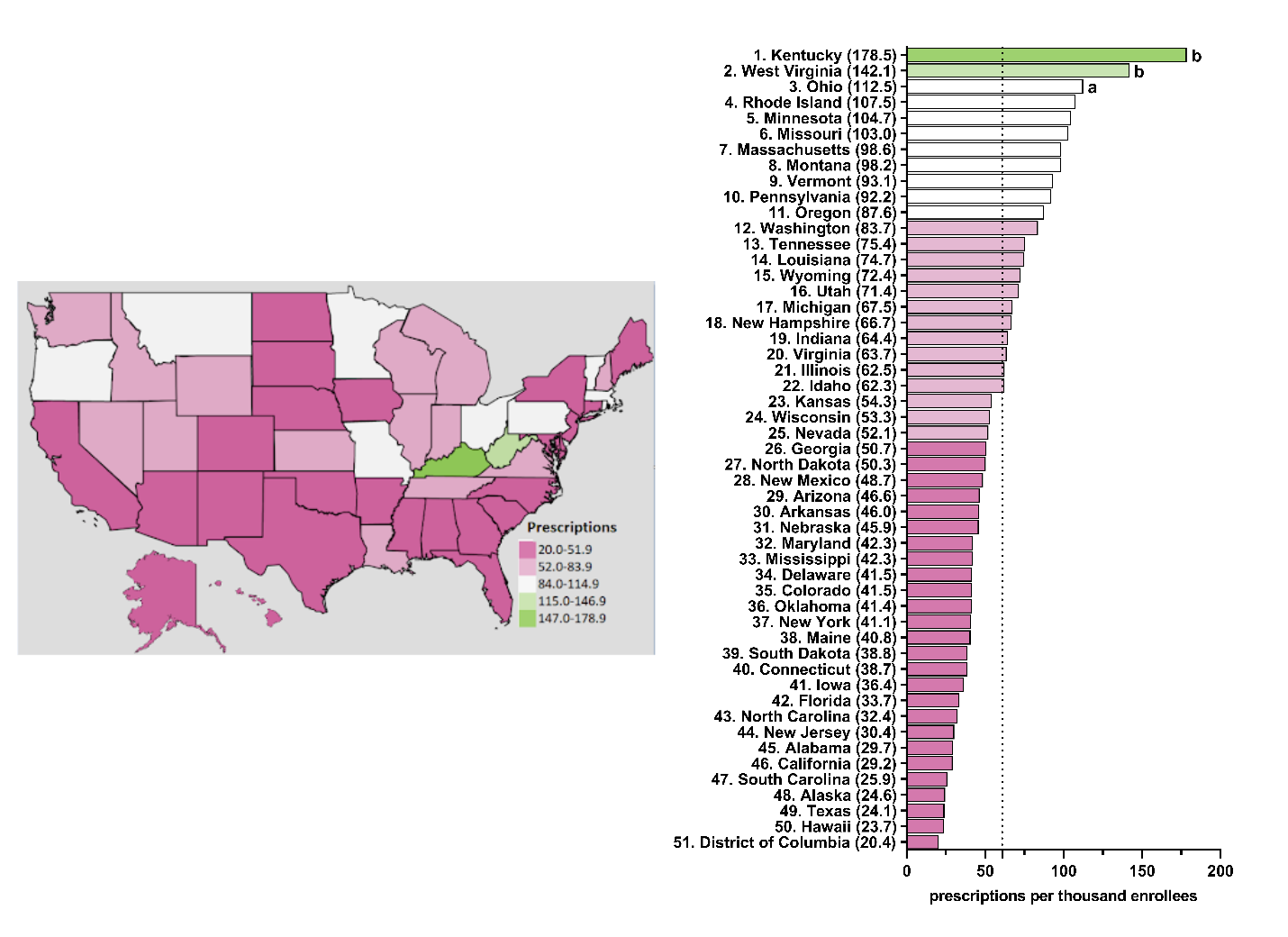


**Supplemental Figure 5.** Citalopram prescriptions per thousand Medicaid enrollees heatmap (left) and population-corrected prescription rate per state (right) in 2019. ^a^ indicates >1.50 SD (29.1) from the mean (55.4). ^b^ indicates >1.96 SD from the mean.


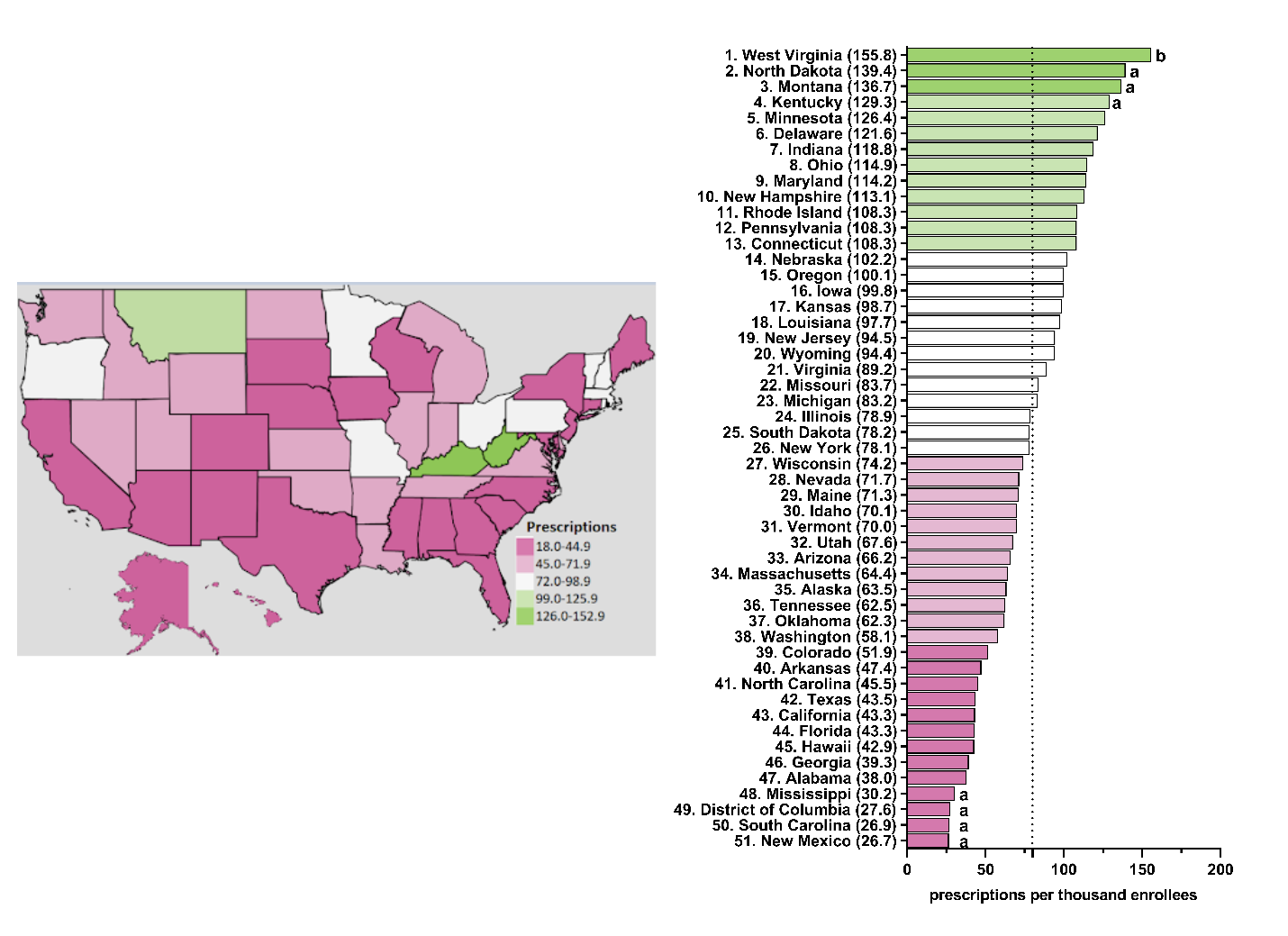


**Supplemental Figure 6.** Escitalopram prescriptions per thousand Medicaid enrollees heatmap (left) and population-corrected prescription rate per state (right) in 2015. ^a^ indicates >1.50 SD (26.2) from the mean (50.0). ^b^ indicates >1.96 SD from the mean.

**
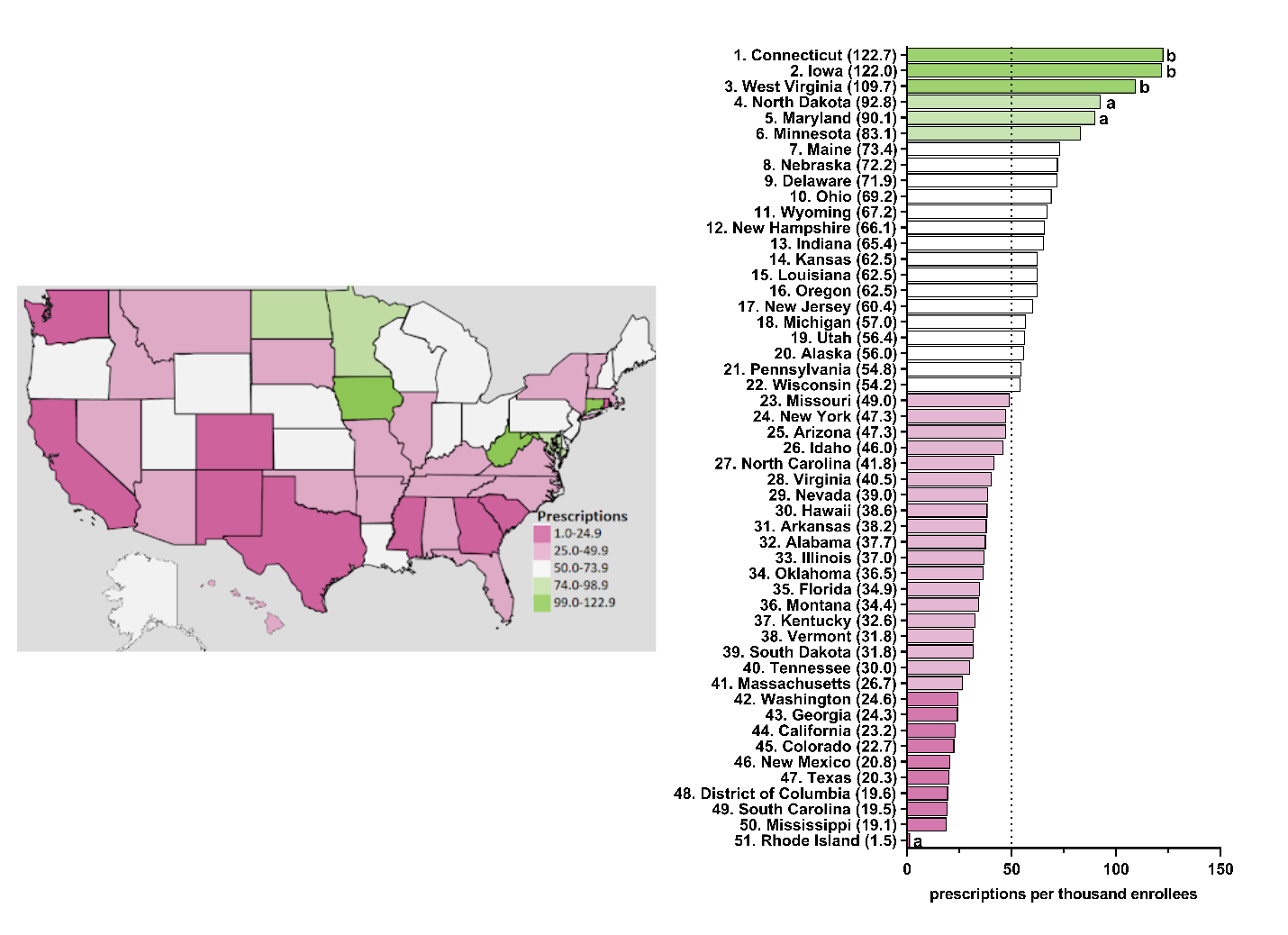
**

**Supplemental Figure 7.** Escitalopram prescriptions per thousand Medicaid enrollees heatmap (left) and population-corrected prescription rate per state (right) in 2016. ^a^ indicates >1.50 SD (27.2) from the mean (58.4). ^b^ indicates >1.96 SD from the mean.

**
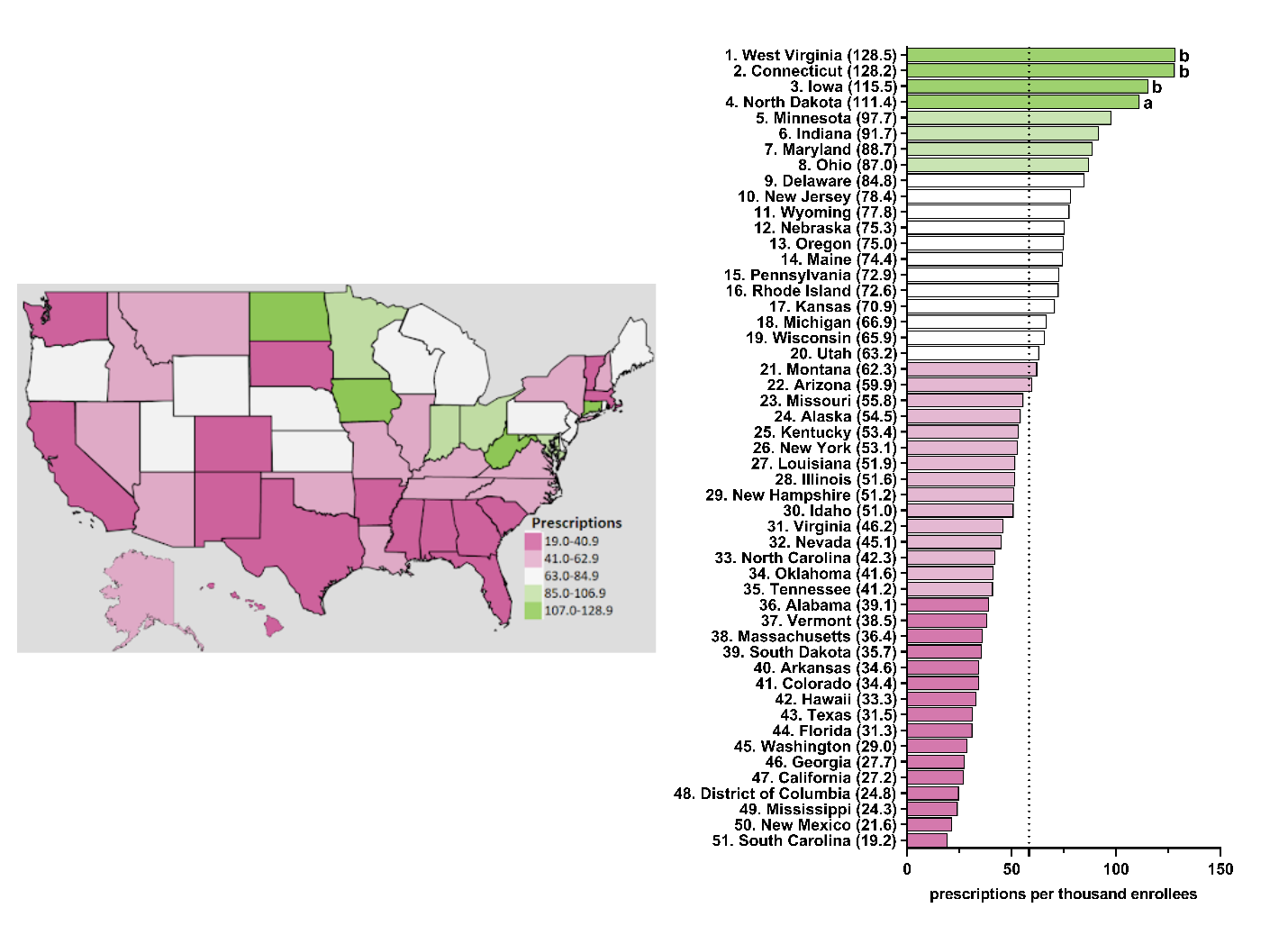
**

**Supplemental Figure 8.** Escitalopram prescriptions per thousand Medicaid enrollees heatmap (left) and population-corrected prescription rate per state (right) in 2017. ^a^ indicates >1.50 SD (27.6) from the mean (66.4). ^b^ indicates >1.96 SD from the mean.

**
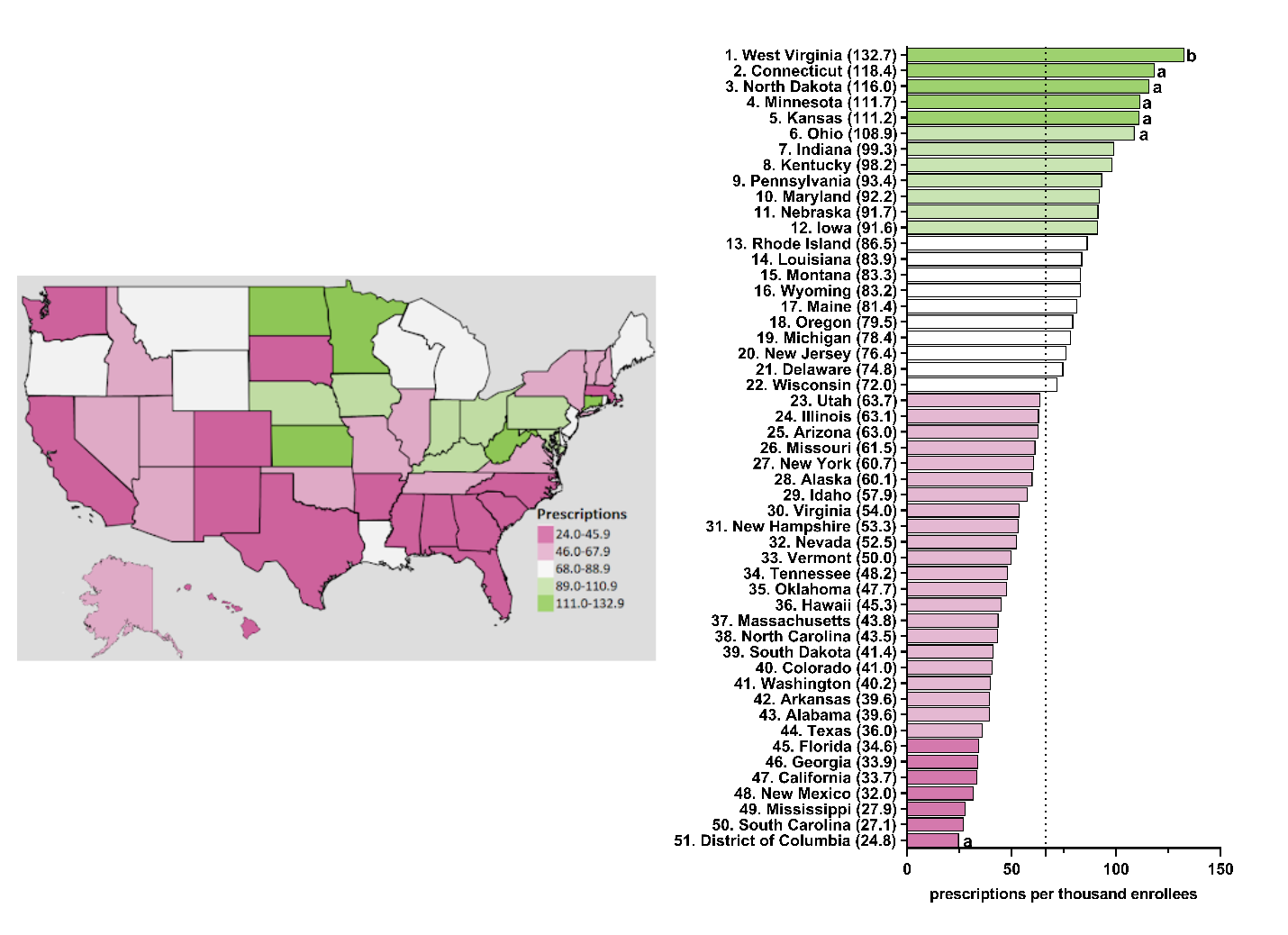
**

**Supplemental Figure 9.** Escitalopram prescriptions per thousand Medicaid enrollees heatmap (left) and population-corrected prescription rate per state (right) in 2018. ^a^ indicates >1.50 SD (28.8) from the mean (73.0). ^b^ indicates >1.96 SD from the mean.

**
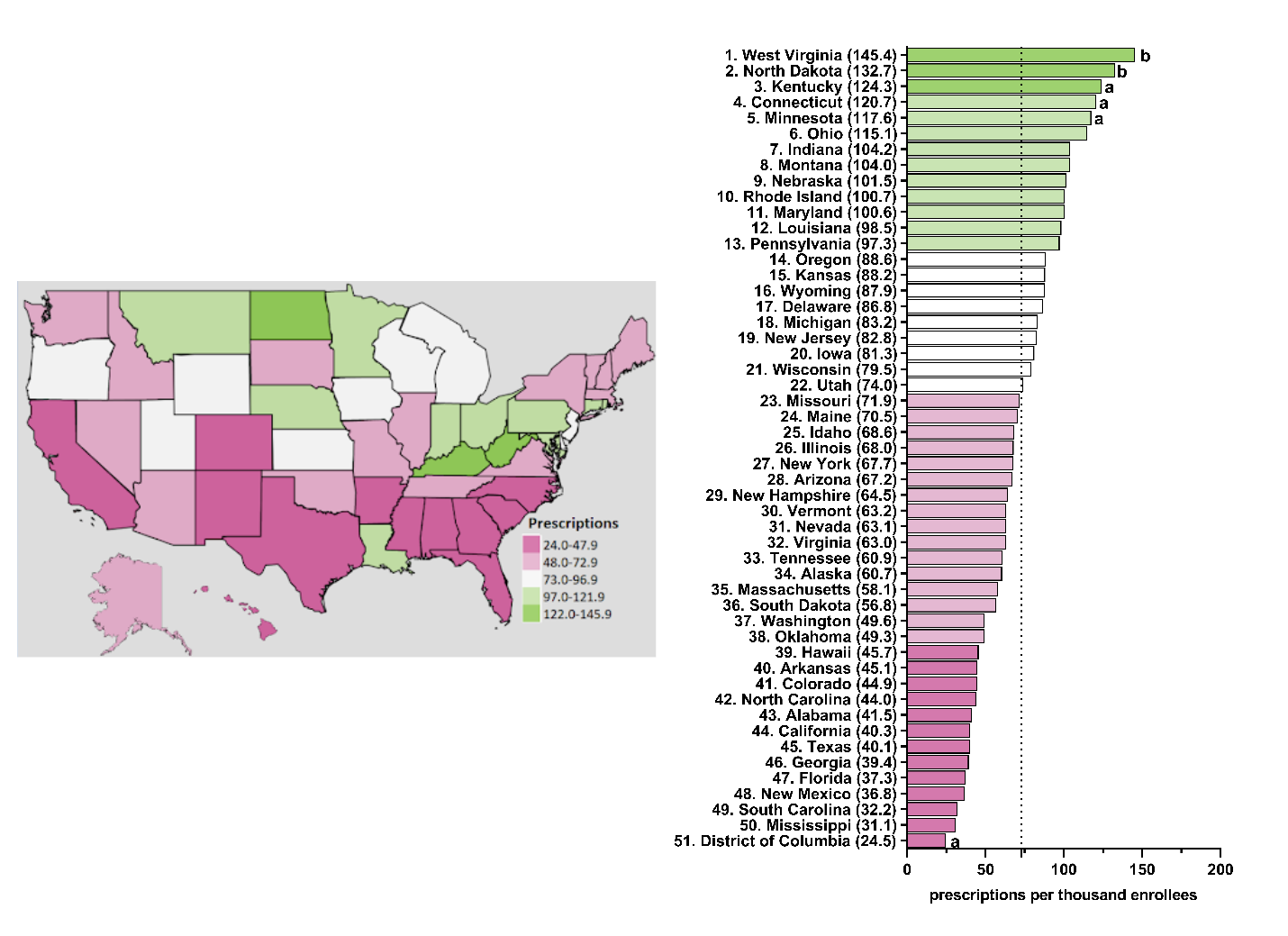
**

**Supplemental Figure 10.** Escitalopram prescriptions per thousand Medicaid enrollees heatmap (left) and population-corrected prescription rate per state (right) in 2019. ^a^ indicates >1.50 SD (32.5) from the mean (80.1). ^b^ indicates >1.96 SD from the mean.

**
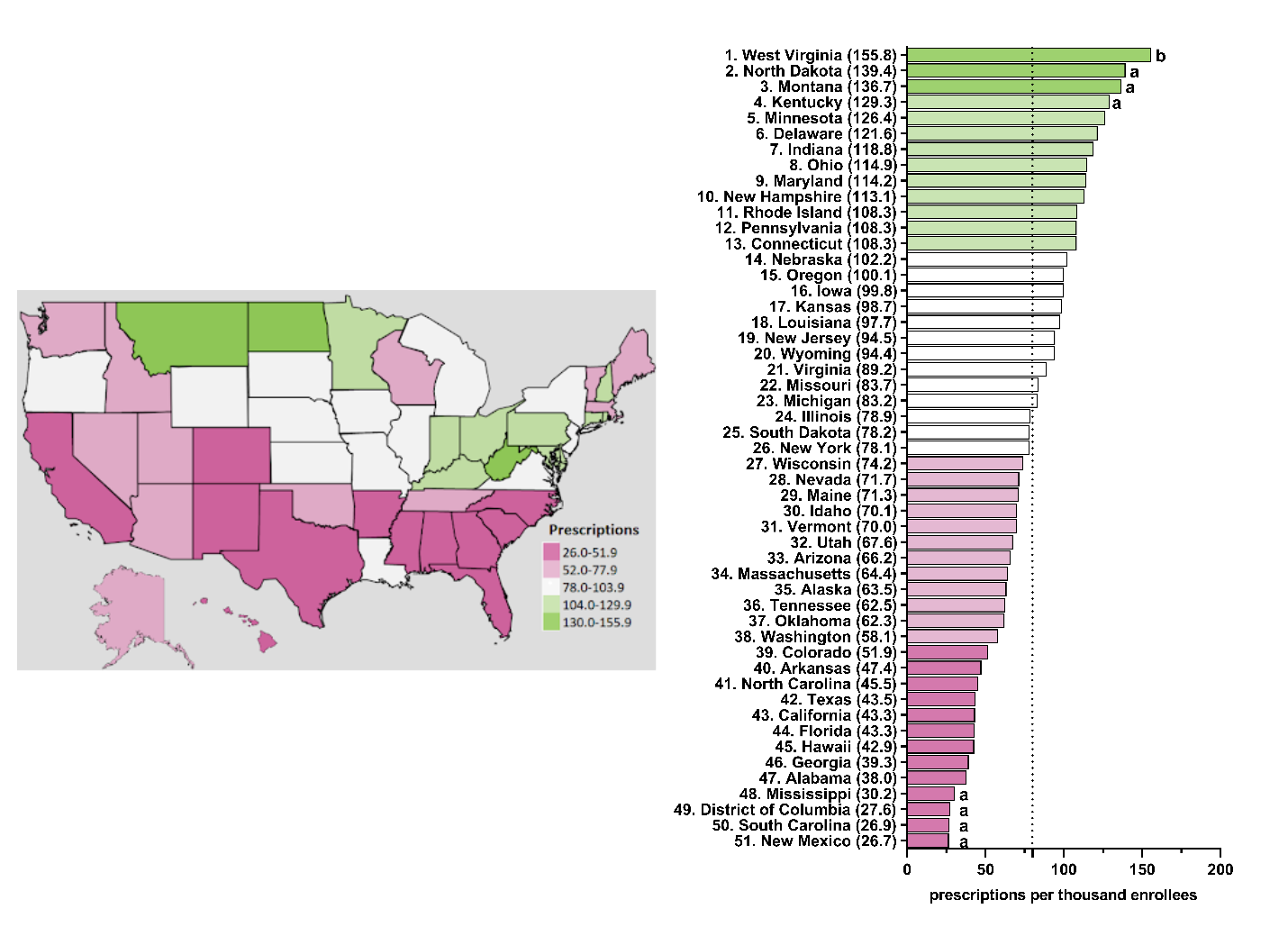
**

**Supplemental Figure 11.** Citalopram prescriptions per thousand Medicare enrollees heatmap (left) and population-corrected prescription rate per state (right) in 2015. ^a^ indicates >1.50 SD (81.0) from the mean (289.7). ^b^ indicates >1.96 SD from the mean.

*
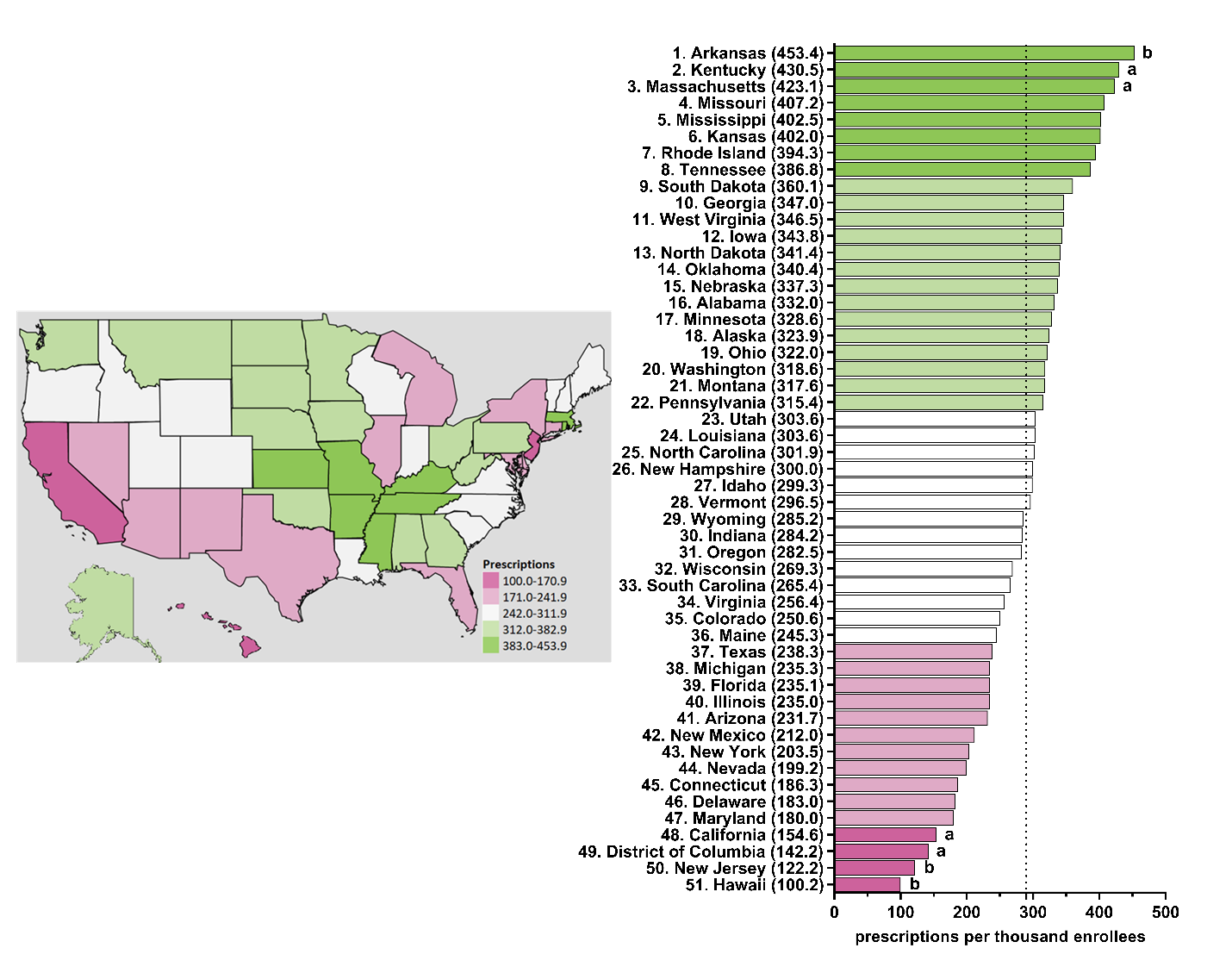
*

**Supplemental Figure 12.** Citalopram prescriptions per thousand Medicare enrollees heatmap (left) and population-corrected prescription rate per state (right) in 2016. ^a^ indicates >1.50 SD (78.7) from the mean (270.9). ^b^ indicates >1.96 SD from the mean.

**
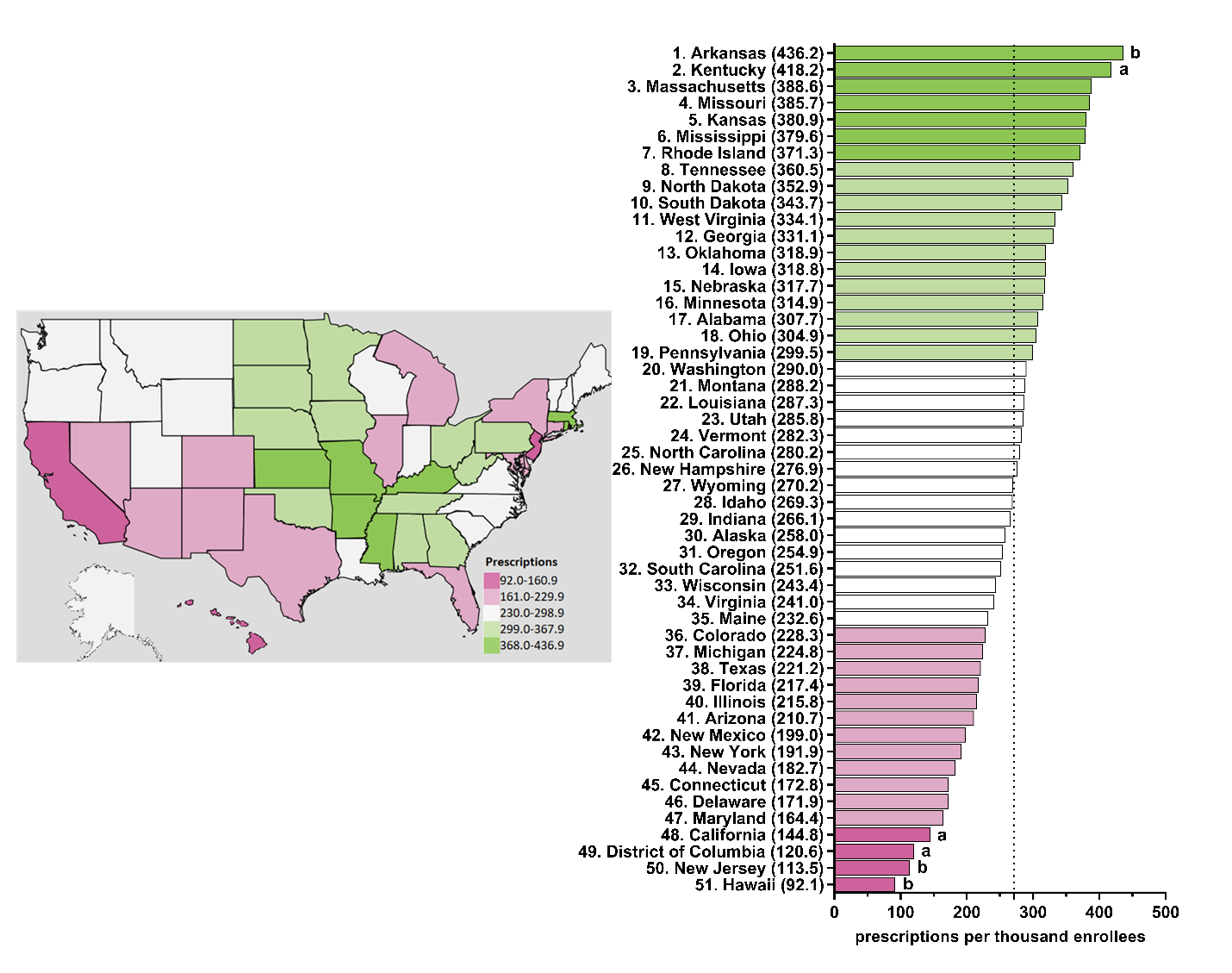
**

**Supplemental Figure 13.** Citalopram prescriptions per thousand Medicare enrollees heatmap (left) and population-corrected prescription rate per state (right) in 2017. ^a^ indicates >1.50 SD (72.7) from the mean (246.6). ^b^ indicates >1.96 SD from the mean.

**
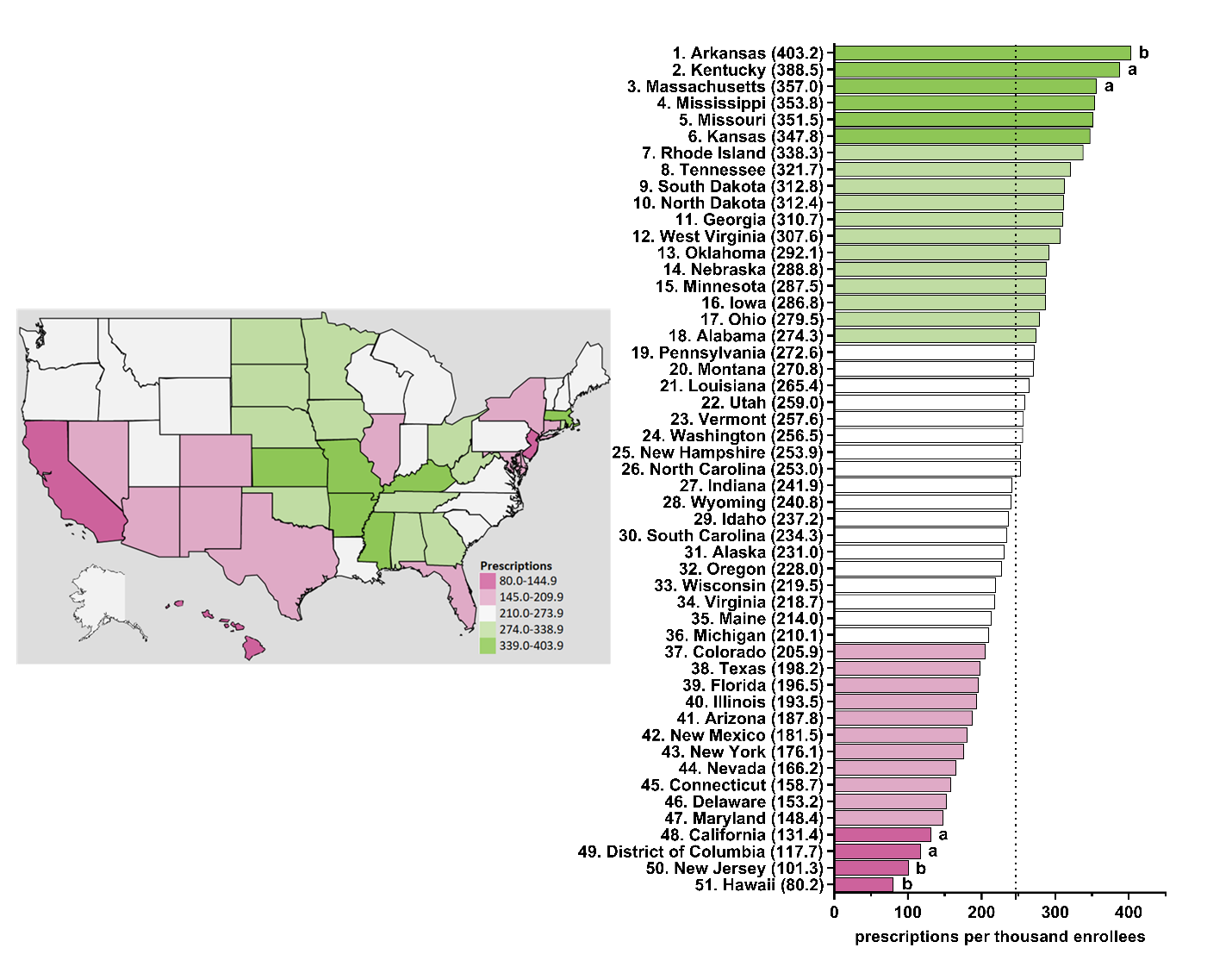
**

**Supplemental Figure 14.** Citalopram prescriptions per thousand Medicare enrollees heatmap (left) and population-corrected prescription rate per state (right) in 2018. ^a^ indicates >1.50 SD (65.4) from the mean (222.7). ^b^ indicates >1.96 SD from the mean.

**
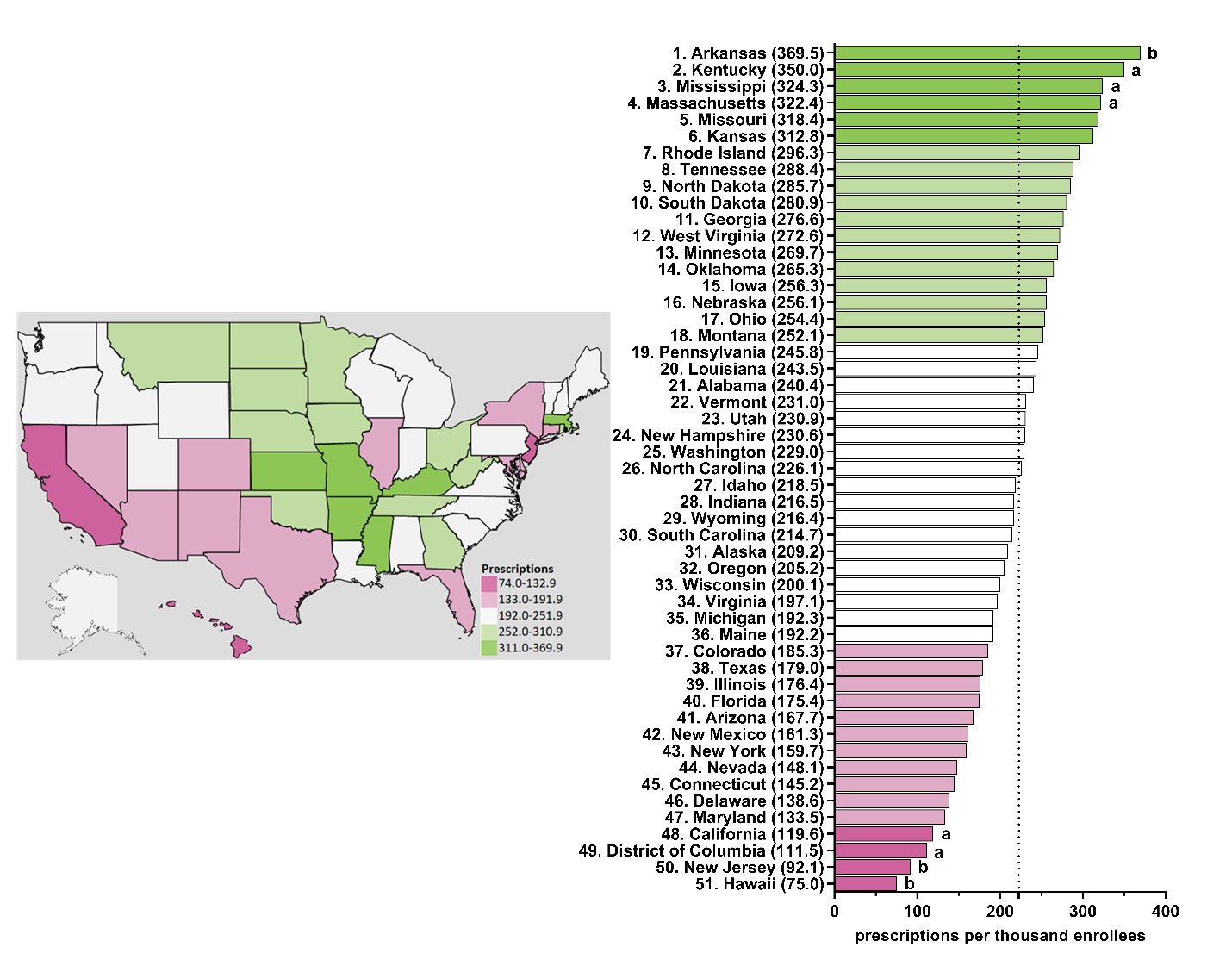
**

**Supplemental Figure 15.** Citalopram prescriptions per thousand Medicare enrollees heatmap (left) and population-corrected prescription rate per state (right) in 2019. ^a^ indicates >1.50 SD (57.6) from the mean (197.7). ^b^ indicates >1.96 SD from the mean.


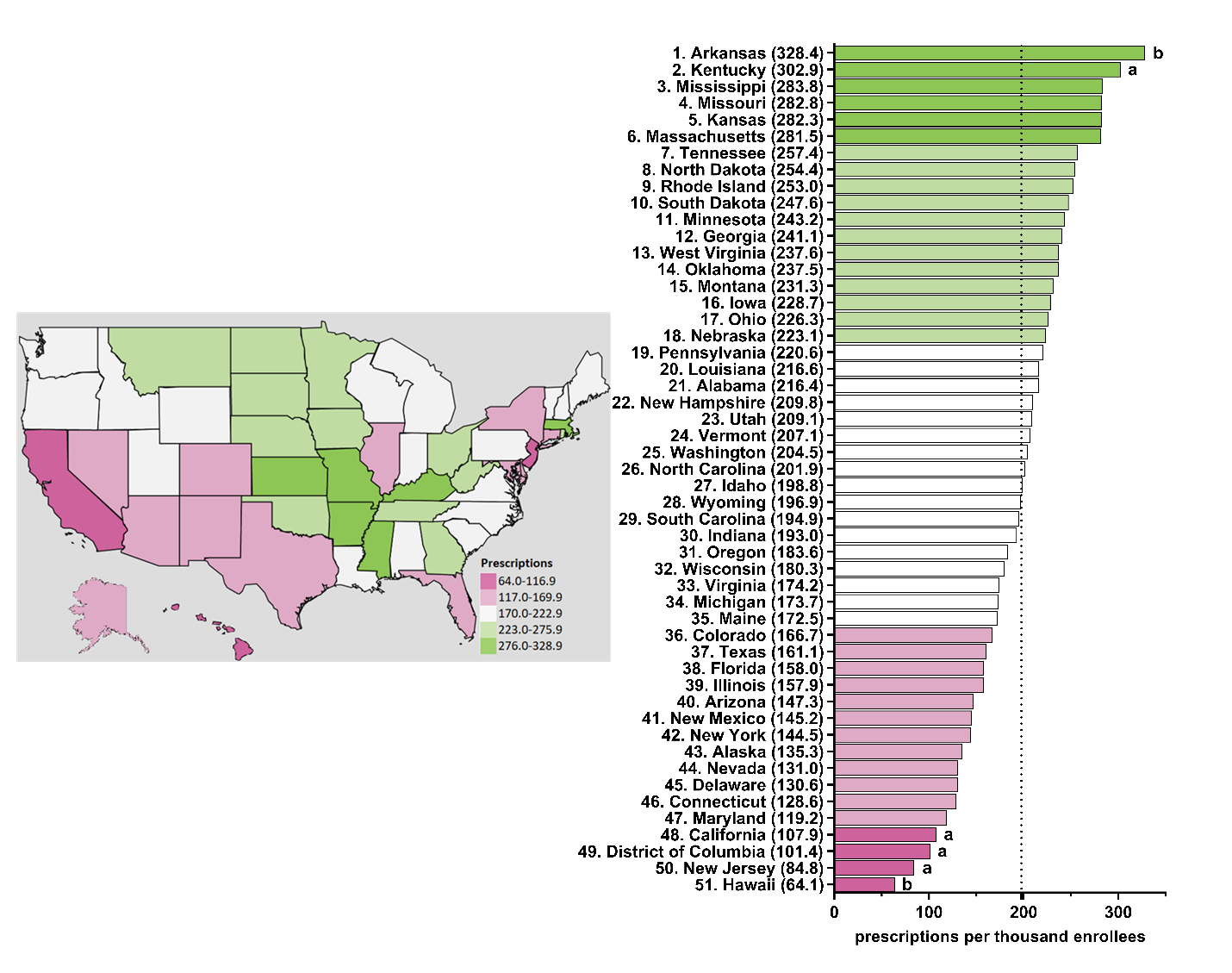


**Supplemental Figure 16.** Escitalopram prescriptions per thousand Medicare enrollees heatmap (left) and population-corrected prescription rate per state (right) in 2015. ^a^ indicates >1.50 SD (55.2) from the mean (210.2). ^b^ indicates >1.96 SD from the mean.

**
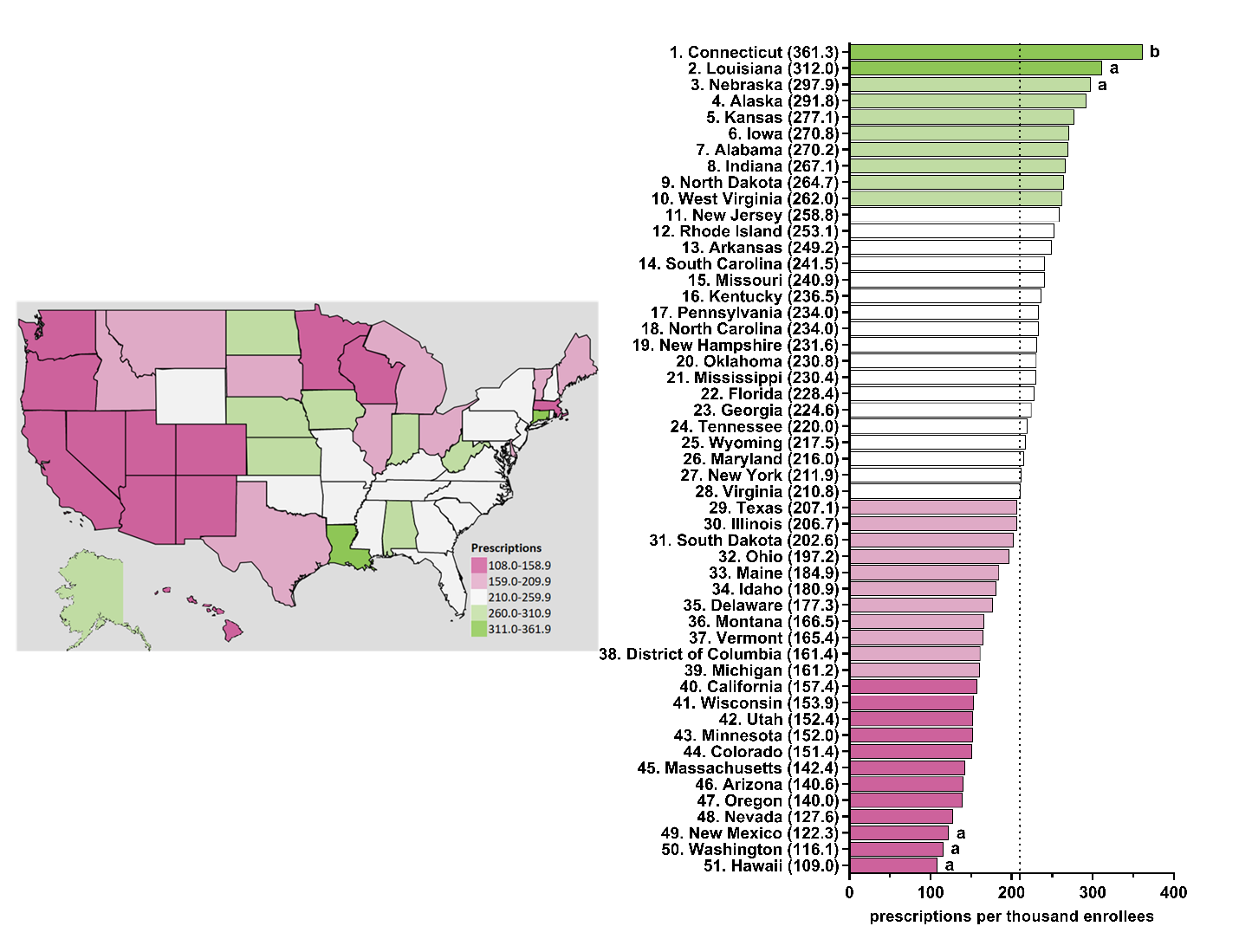
**

**Supplemental Figure 17.** Escitalopram prescriptions per thousand Medicare enrollees heatmap (left) and population-corrected prescription rate per state (right) in 2016. ^a^ indicates >1.50 SD (56.3) from the mean (218.9). ^b^ indicates >1.96 SD from the mean.

**
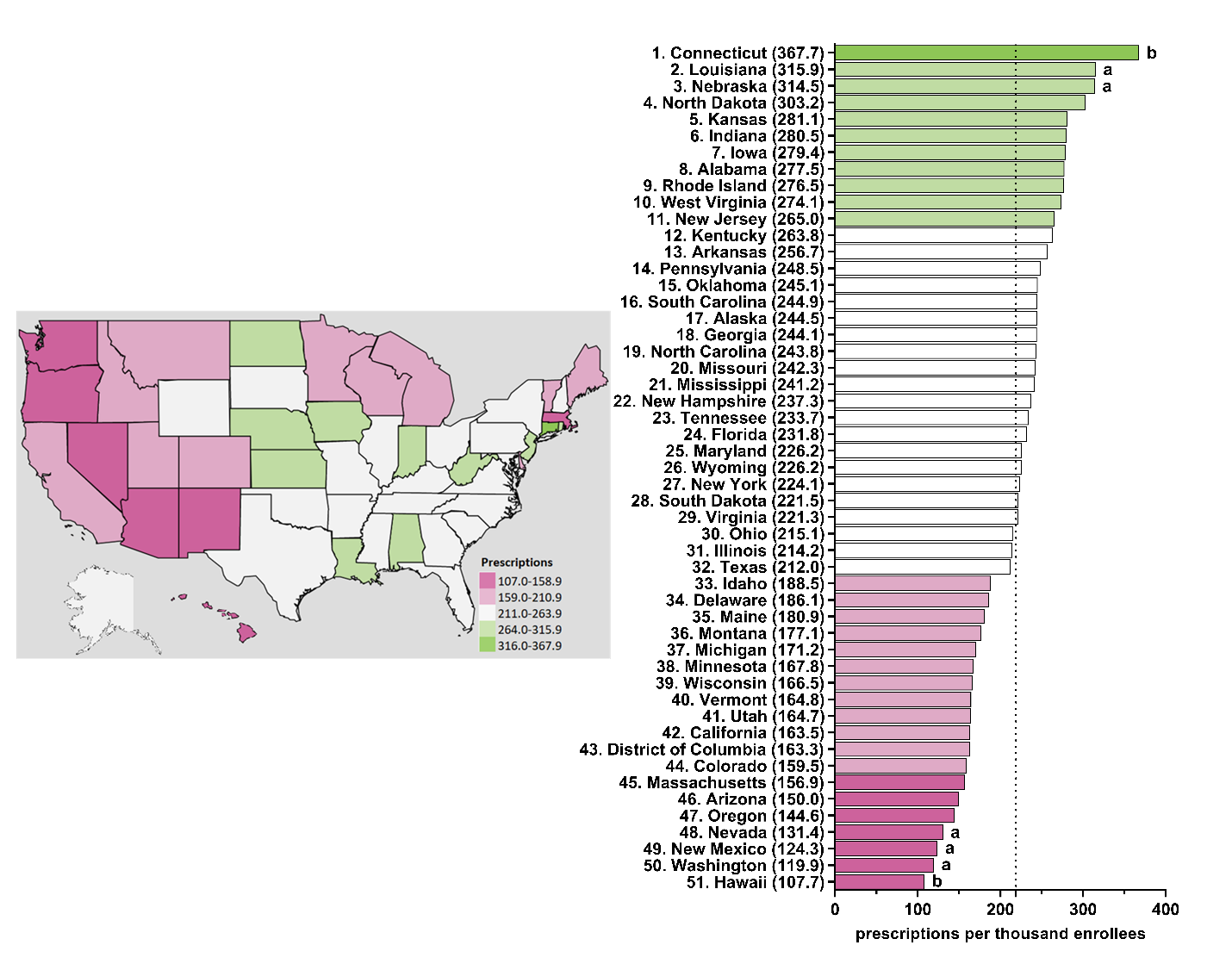
**

**Supplemental Figure 18.** Escitalopram prescriptions per thousand Medicare enrollees heatmap (left) and population-corrected prescription rate per state (right) in 2017. ^a^ indicates >1.50 SD (56.2) from the mean (222.5). ^b^ indicates >1.96 SD from the mean.

**
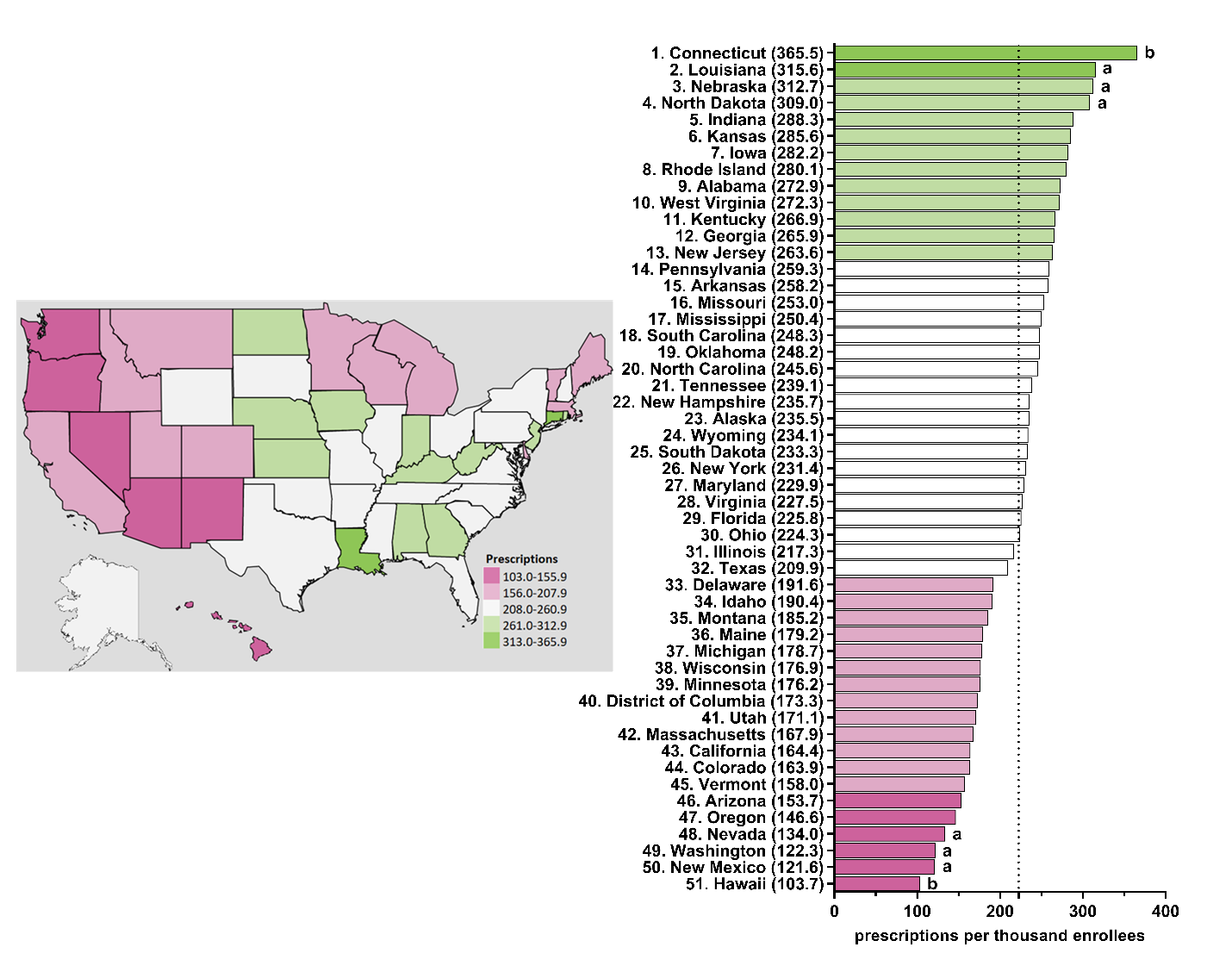
**

**Supplemental Figure 19.** Escitalopram prescriptions per thousand Medicare enrollees heatmap (left) and population-corrected prescription rate per state (right) in 2018. ^a^ indicates >1.50 SD (54.2) from the mean (222.1). ^b^ indicates >1.96 SD from the mean.


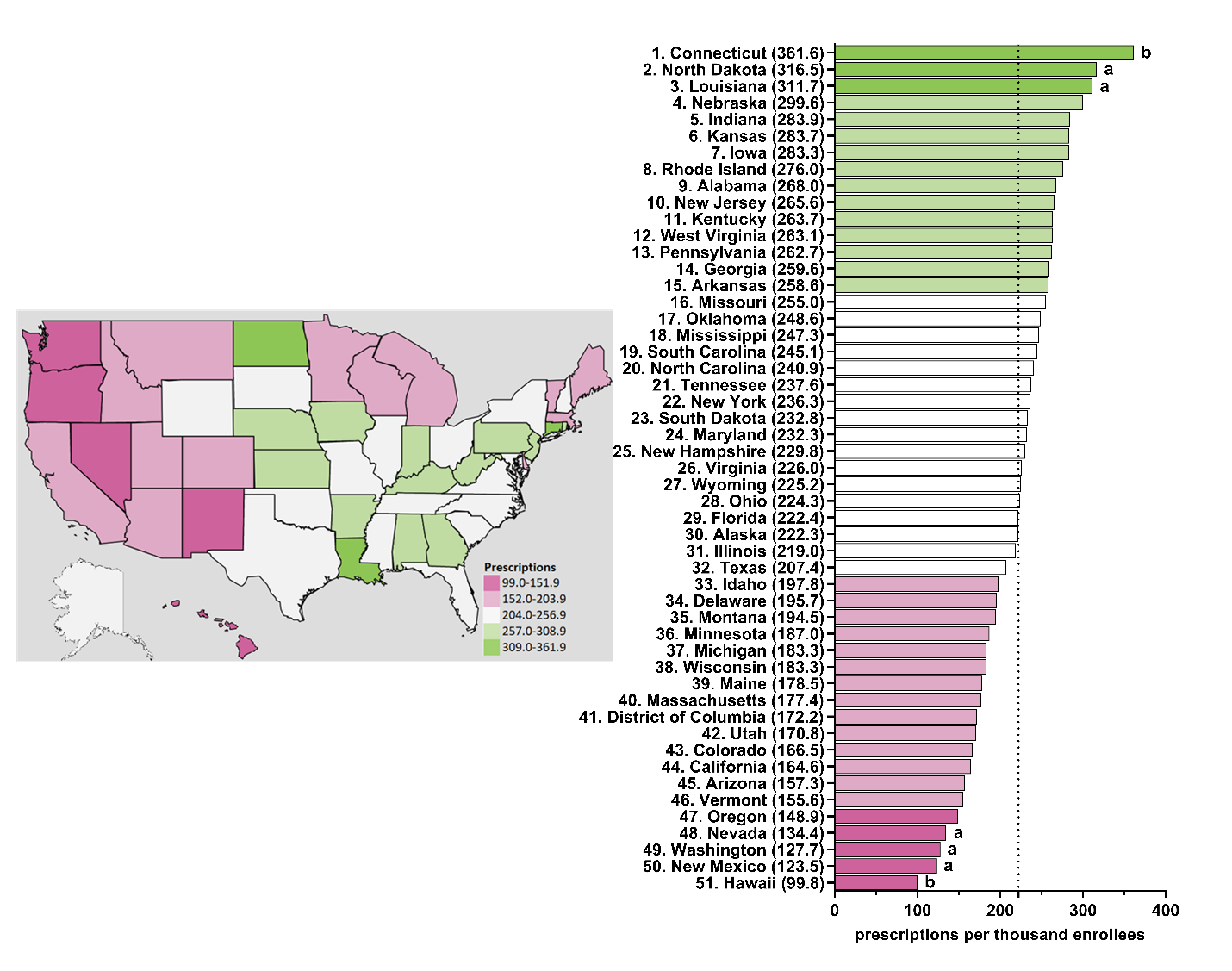


**Supplemental Figure 20.** Escitalopram prescriptions per thousand Medicare enrollees heatmap (left) and population-corrected prescription rate per state (right) in 2019. ^a^ indicates >1.50 SD (52.0) from the mean (218.9). ^b^ indicates >1.96 SD from the mean.


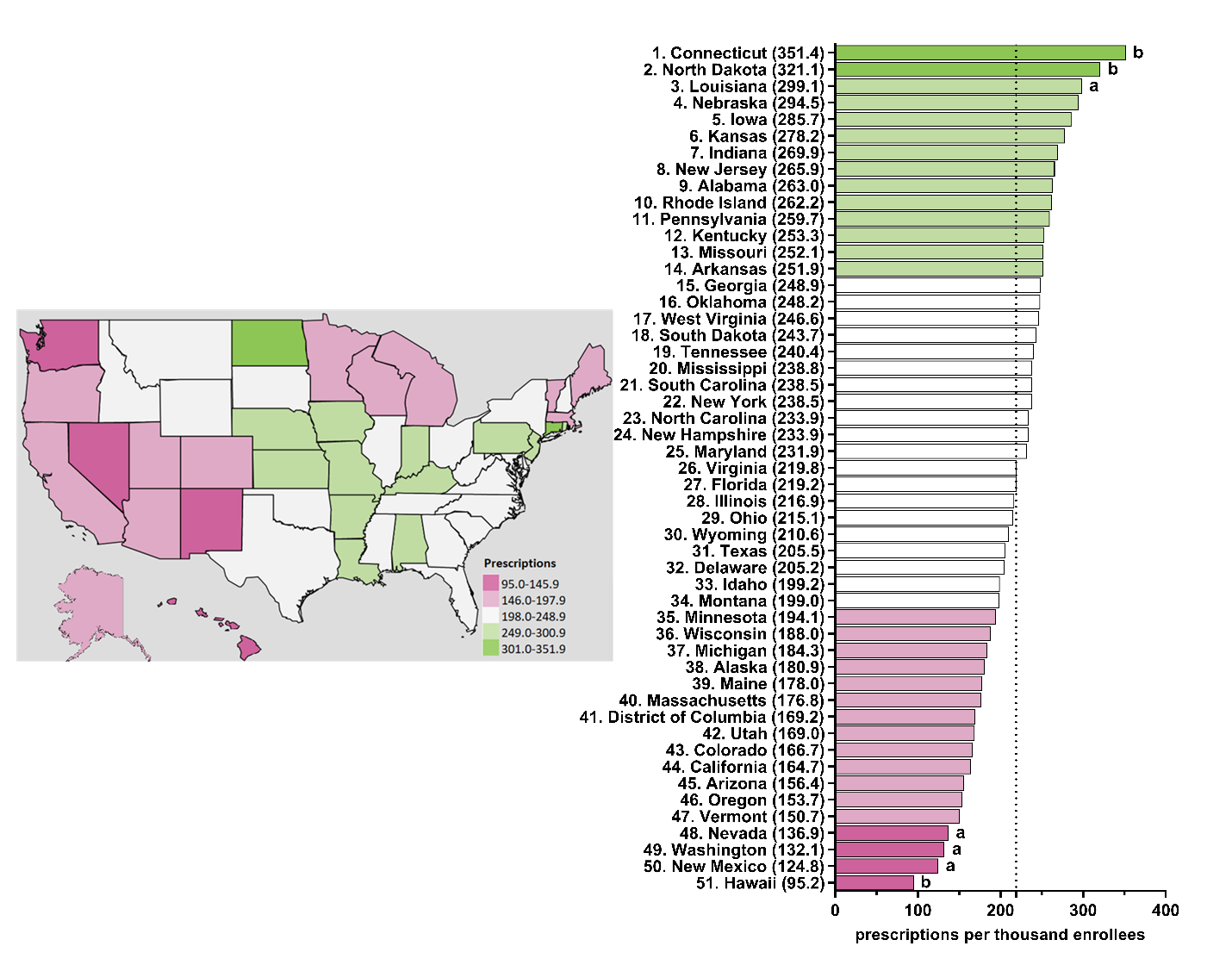


**Supplemental Figure 21**. Matrix of Pearson’s correlation coefficients between population-corrected number of prescriptions of citalopram and escitalopram within the Medicaid and Medicare systems for 2015 (*N*=51: 50 states and D.C.). * indicates *p*≤0.05, ** indicates *p*≤0.01, and *** *p*≤0.001.


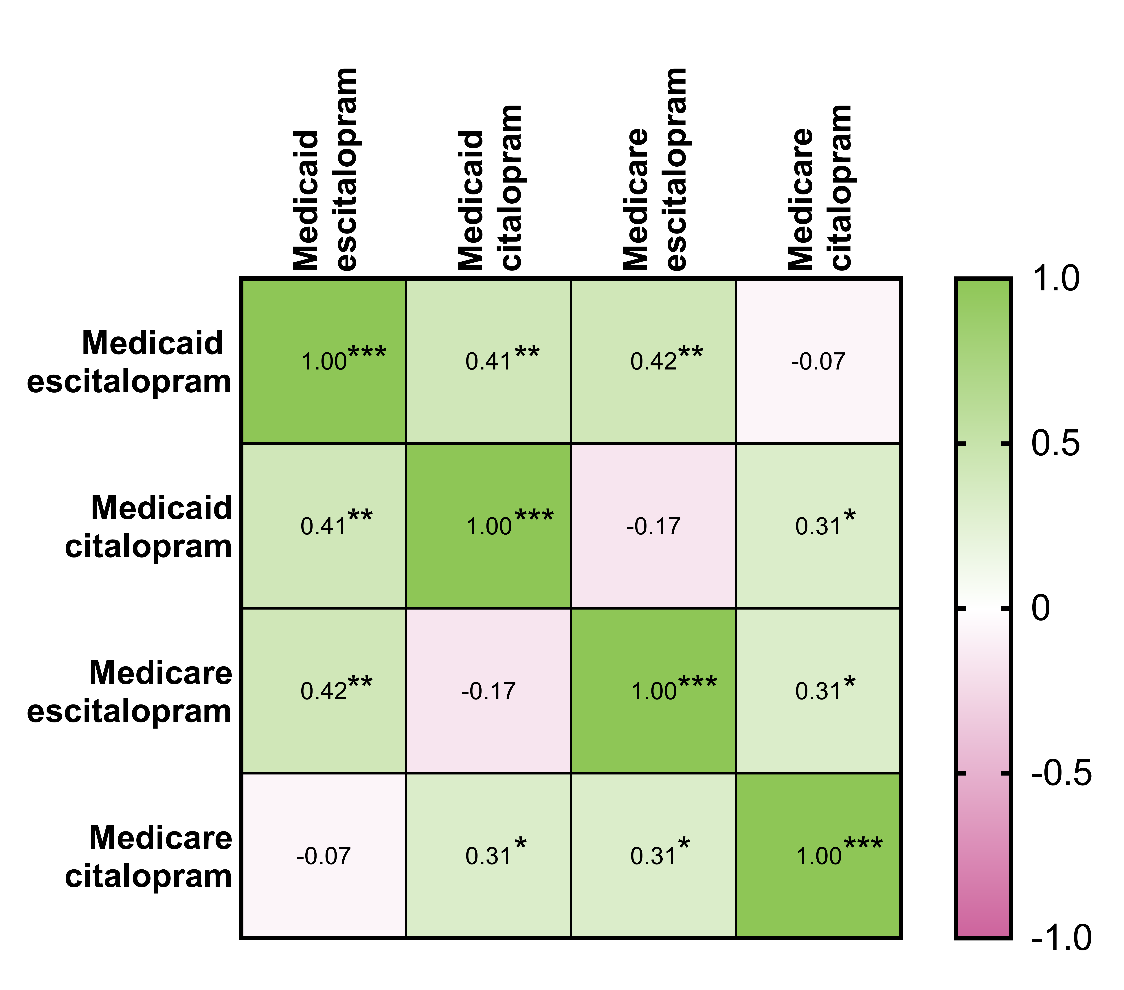


**Supplemental Figure 22**. Matrix of Pearson’s correlation coefficients between population-corrected number of prescriptions of citalopram and escitalopram within the Medicaid and Medicare systems for 2016 (*N*=51: 50 states and D.C.). * indicates *p*≤0.05, ** indicates *p*≤0.01, and *** *p*≤0.001.


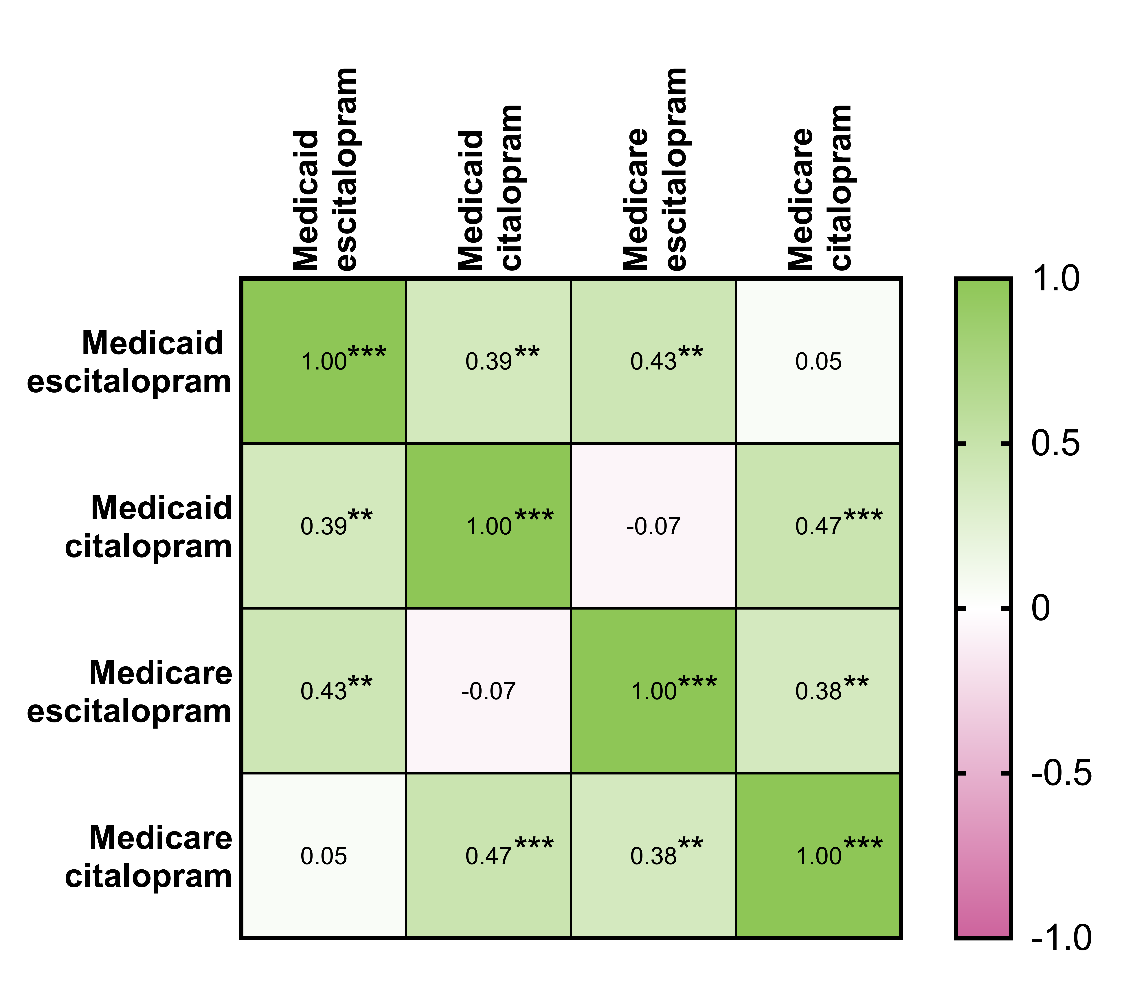


**Supplemental Figure 23**. Matrix of Pearson’s correlation coefficients between population-corrected number of prescriptions of citalopram and escitalopram within the Medicaid and Medicare systems for 2017 (*N*=51: 50 states and D.C.). * indicates *p*≤0.05, ** indicates *p*≤0.01, and *** *p*≤0.001.


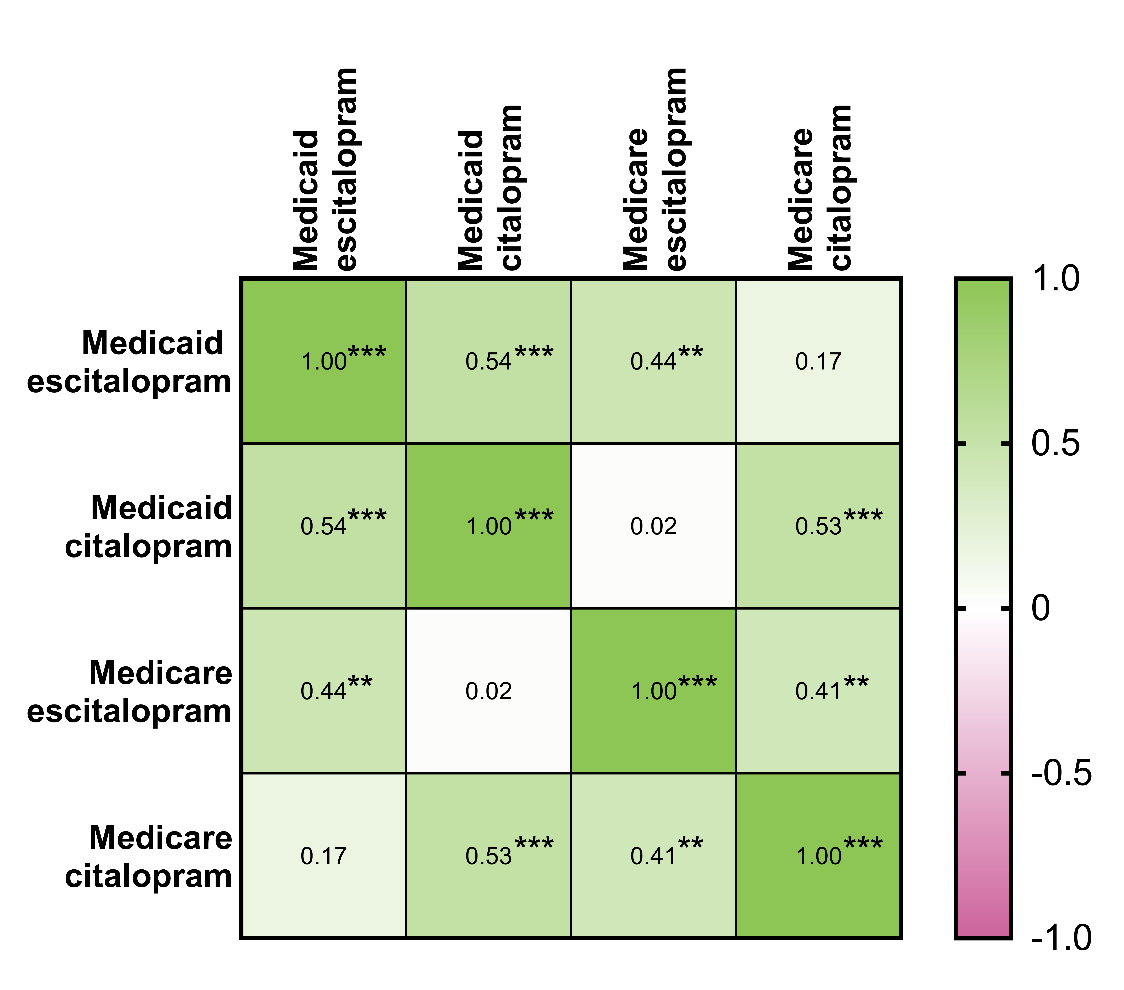


**Supplemental Figure 24**. Matrix of Pearson’s correlation coefficients between population-corrected number of prescriptions of citalopram and escitalopram within the Medicaid and Medicare systems for 2018 (*N*=51: 50 states and D.C.). * indicates *p*≤0.05, ** indicates *p*≤0.01, and *** *p*≤0.001.


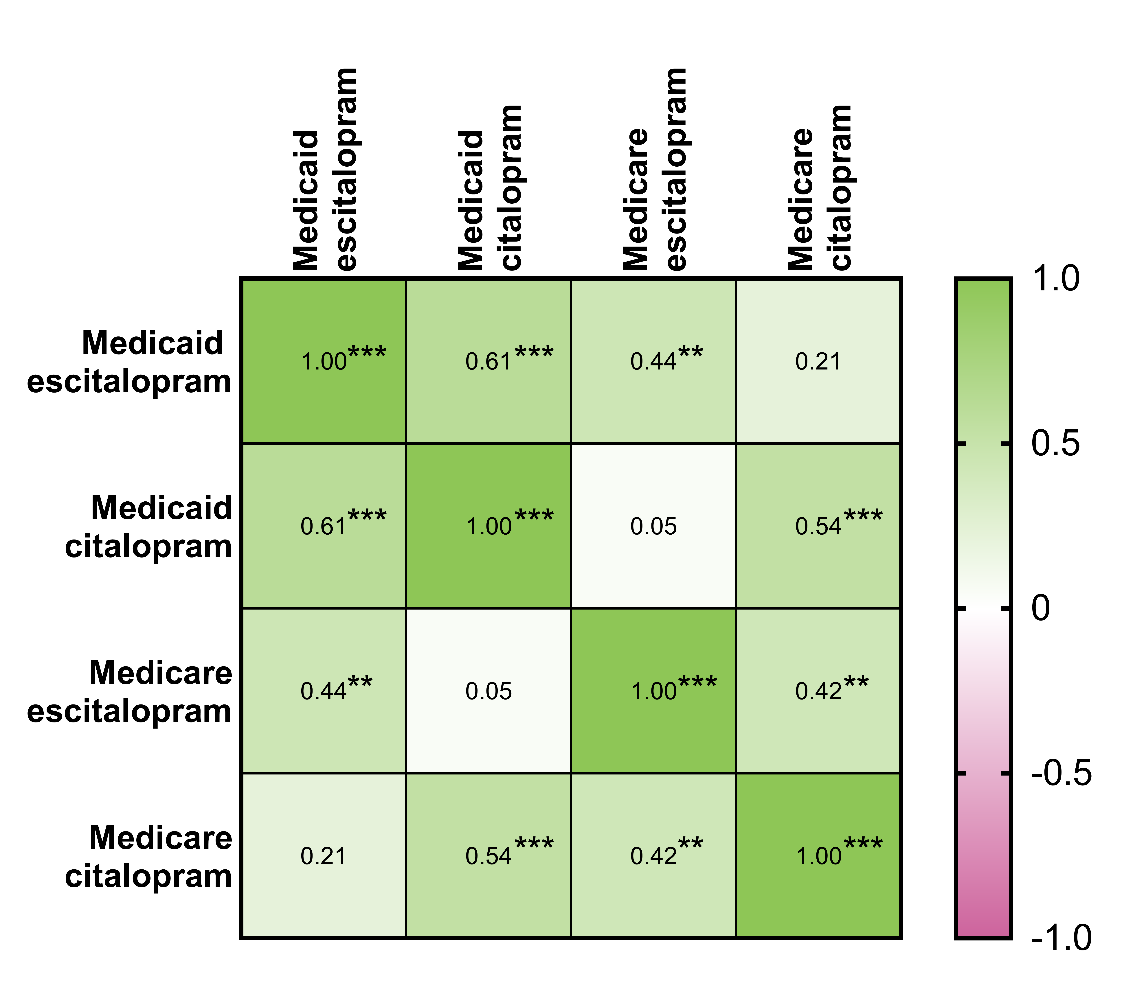


**Supplemental Figure 25**. Matrix of Pearson’s correlation coefficients between population-corrected number of prescriptions of citalopram and escitalopram within the Medicaid and Medicare systems for 2019 (*N*=51: 50 states and D.C.). * indicates *p*≤0.05, ** indicates *p*≤0.01, and *** *p*≤0.001.


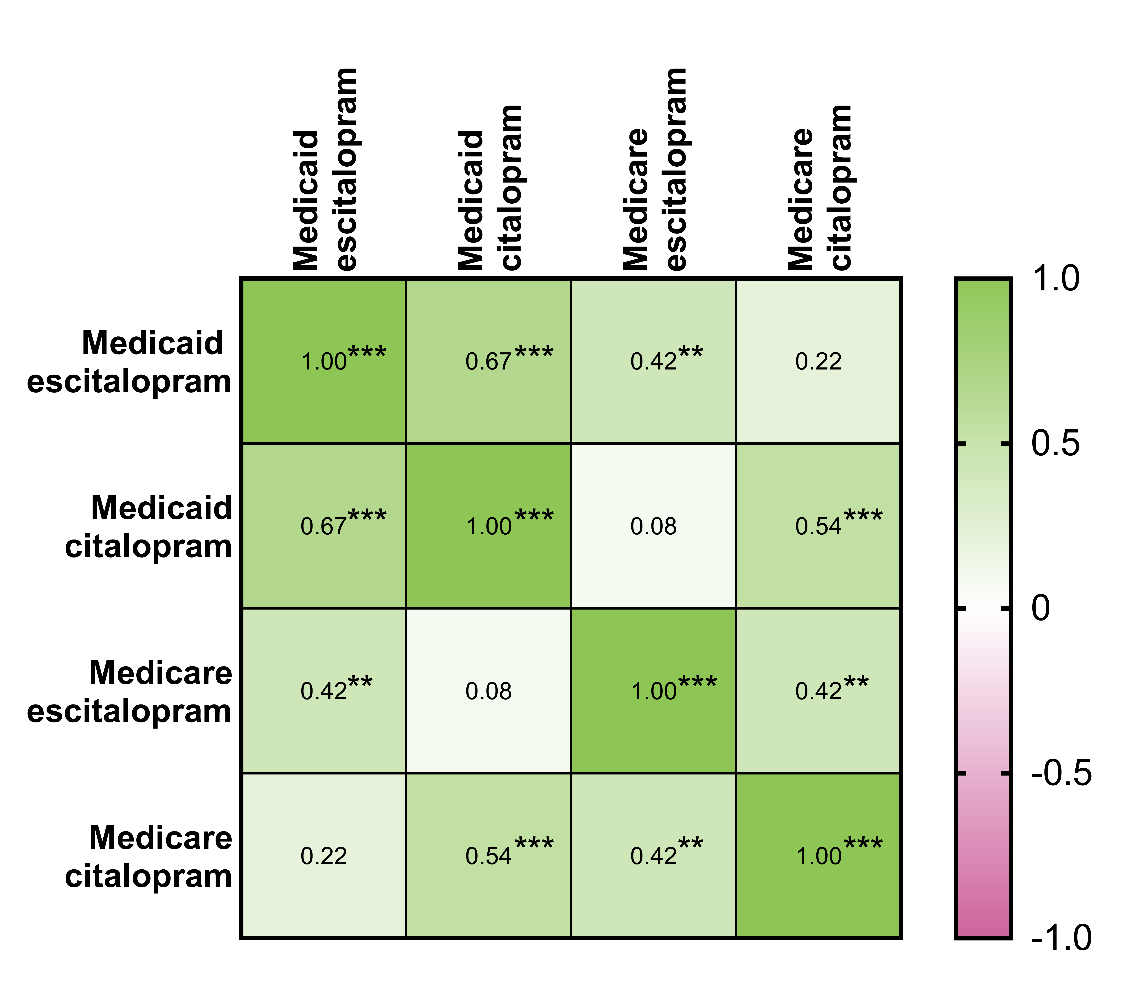

Supplement: Supplementary file 1 [file SupplementaryFile1.docx]
